# A meta‐analytic review of the relationship between racial discrimination and alcohol and other drug use outcomes in minoritised racial/ethnic groups

**DOI:** 10.1111/add.70131

**Published:** 2025-07-16

**Authors:** Evie Gates, Matthew Cant, Rebecca Elliott, Patricia Irizar, Christopher J. Armitage

**Affiliations:** ^1^ Division of Psychology and Mental Health University of Manchester, Faculty of Biology, Medicine and Health, School of Health Sciences Manchester UK; ^2^ School of Psychology Liverpool John Moores University Liverpool UK

**Keywords:** alcohol use, alcohol use disorder, ethnic minority, health inequality, racial discrimination, racism, substance use, substance use disorder

## Abstract

**Aims:**

To measure the associations between racial discrimination and distinct alcohol and other drug use outcomes in minoritised racial/ethnic groups and to explore the moderating roles of demographic and methodological characteristics.

**Methods:**

Quantitative studies including racial discrimination as an exposure (both binary and continuous), an alcohol and/or other drug use outcome and a minoritised racial/ethnic sample were identified via database, citation and journal searching. 130 studies contributing 273 effect sizes, across seventeen distinct outcomes, were included in this analysis. Random‐effects meta‐analytic models were implemented. Moderation effects were explored using subgroup analyses.

**Results:**

Racial discrimination was positively associated with sixteen alcohol and other drug use outcomes. The strongest associations were observed for at‐risk/hazardous alcohol use [*r* = 0.24, 95% confidence interval (CI) = 0.17–0.3, I^2^ = 94.8%, m = 29, *n* = 9445], at‐risk/hazardous cannabis use (*r* = 0.24, 95% CI = 0.18–0.29, I^2^ = 0%, m = 4, *n* = 462) and substance use disorder (*r* = 0.25, 95% CI = 0.14–0.36, I^2^ = 97.7%, m = 5, *n* = 21 051). Considerable heterogeneity was observed across fourteen outcomes (I^2^ = 69.5%–97.7%). Concerning tobacco use, Indigenous North Americans had the largest effect (*r* = 0.27, 95% CI = 0.2–0.35, I^2^ = 0%, m = 2, *n* = 529), followed by Black Americans (*r* = 0.06, 95% CI = 0.01–0.12, I^2^ = 81.7%, m = 7, *n* = 5409). Little evidence for an association was found for Latinxs (*r* = 0.06, 95% CI = –0.02 to 0.14, I^2^ = 89.2%, m = 3, *n* = 5404) or Asian Americans (*r* = –0.18, 95% CI = –0.8 to 0.43, I^2^ = 99%, m = 2, *n* = 572). Regarding composite substance use, Indigenous North Americans had the strongest associations (*r* = 0.29, 95% CI = 0.23–0.35, I^2^ = 0%, m = 3, *n* = 778), followed by Black Americans (*r* = 0.13, 95% CI = 0.09–0.18, I^2^ = 62.8%, m = 7, *n* = 5981) and then Latinxs (*r* = 0.07, 95% CI = –0.17 to 0.31, I^2^ = 91.3%, m = 4, *n* = 1646). Concerning alcohol use problems, younger samples produced stronger associations (*r* = 0.28, 95% CI = 0.17–0.38, I^2^ = 38.8%, m = 3, *n* = 483), while older samples showed larger effects in six other outcomes (*rs* = 0.13–0.26). Regarding at‐risk/hazardous alcohol use and alcohol use problems/consequences, cross‐sectional studies (*rs* = 0.23–0.24) produced stronger associations than longitudinal studies (*rs* = 0.13–0.14). Concerning tobacco and illicit substance use, the strongest associations were identified for lifetime exposure (*rs* = 0.18–0.32).

**Conclusions:**

Racial discrimination appears to be a consistent correlate of multiple alcohol and other drug use outcomes in minoritised racial/ethnic groups, predominantly based in the United States, yet the magnitude of these associations differs across outcomes. Demographic and methodological characteristics somewhat moderate these associations.

## INTRODUCTION

Race and ethnicity are terms often used interchangeably, yet they are distinct but related constructs. Race is a social construct with no meaningful biological basis that has historically and contemporaneously been used to justify the dominion of the dominant racial group [1, 2, 3, 4]. Despite no universal definition, there is some consensus that race refers to grouping people based on shared ancestry and/or phenotype [5, 6, 7, 8, 9]. Ethnicity is also a multi‐dimensional social construct that pertains to shared cultural, ancestral, linguistic, religious and physical characteristics [10, 11, 12, 13, 14]. The determination of which racial and ethnic groups are minoritised differs widely across countries because of their varied historical, social and political contexts. The present study, however, defines minoritised racial and ethnic groups as those that are either numerically smaller than the rest of the population, hold a non‐dominant political, social or economic position in society or have an ethnicity, religion or language that differs from the majority [15].

Minoritised racial and ethnic groups have been reported to be at increased risk for alcohol and other drug use (AOD) at different stages of the life course [16, 17, 18, 19]. They may also be less likely to ‘age out’ of use [20] and more likely to experience negative consequences of use [21]. Exposure to racial discrimination, which can be defined as unfair treatment attributed to one's ethnicity, race or culture of origin [22, 23, 24], may help explain these increased risks [25, 26, 27]. It has been posited that minoritised racial/ethnic groups may engage in AOD to cope with the stress of discrimination [28, 29]. This is a particularly pertinent theory, because it contextualises racism within pre‐existing models of AOD, such as stress‐coping theory [30], tension‐reduction models [31], motivation models [32] and the self‐medication hypothesis [33]. A 2017 meta‐analysis of six effect sizes, however, failed to show a significant association between racial discrimination and substance use in United States (US)‐based minoritised racial/ethnic groups [34]. Yet later meta‐analyses have reported significant associations (*r* = 0.16) (*r* = 0.13) in minoritised racial/ethnic groups residing in the United States and internationally [35, 36], although these effect sizes are smaller than anticipated.

A closer examination of these meta‐analyses reveals important limitations. First, they report the association between racial discrimination and a composite measure of substance use, capturing different AOD outcomes within one generic ‘substance use’ outcome [34, 35, 36]. This method assumes that these outcomes are equivalent. In the US literature, some studies have reported little variation in the association between racial discrimination and distinct AOD outcomes. For example, comparable effect sizes have been reported across tobacco, cannabis, alcohol, prescription opioid and heavy alcohol use [37, 38]. Other research, however, has observed that racial discrimination is differentially associated with distinct types and patterns of AOD, including polysubstance use, dual substance use, binge drinking, alcohol use consequences, cigarette use, alcohol use, cannabis use and prescription substance use [39, 40, 41, 42, 43].

A second potential limitation of previous meta‐analyses is the lack of moderation analyses across distinct AOD outcomes, as there is some evidence from the United States to suggest that the racial discrimination‐AOD relationships vary by race/ethnicity, gender and age. For example, the association between racial discrimination and heavy alcohol use has been documented to be stronger in African Americans and Hispanics, compared to Chinese Americans [44]. Variability by age and gender has not been extensively studied, as they are typically used as covariates, obscuring their potentially moderating effects. However, Assari and colleagues [45] observed that exposure to racial discrimination in adolescence predicts increased cannabis use in adulthood in African American males, but decreased use in females. Gender differences have also been observed in Latinxs, where racial discrimination is more strongly associated with drug and alcohol abuse in males [46]. Moreover, experiences of racial discrimination in African Americans have been demonstrated to have a stronger association with regular smoking in respondents below age 45 [47]. Similar findings are reported for associations between racial discrimination and substance use disorder in African American, Hispanic and Asian participants [48].

### Current study

The current study aims to quantitatively synthesise the literature on relationships between racial discrimination and AOD outcomes in minoritised racial/ethnic groups internationally. Considering the findings from previous meta‐analyses, this study intends to (1) determine the strength of associations between racial discrimination and distinct AOD outcomes in minoritised racial/ethnic groups; and (2) assess whether these associations are modified by race/ethnicity, age, gender, exposure timing and study design. To our knowledge, this is the first meta‐analysis to assess the role of racial discrimination across distinct AOD outcomes, within multiple minoritised racial/ethnic groups and across numerous countries. Therefore, it intends to provide critical insights into the role of racial discrimination in AOD, with the aim that this knowledge can guide intervention and prevention strategies, policy and educational practices.

## METHODOLOGY

This review was registered on PROSPERO, the systematic review registry (ID: CRD42022381762). This study was also conducted and reported in line with the Preferred Reporting Items for Systematic Reviews and Meta‐analysis (PRISMA) [49].

### Search strategy

**FIGURE 1:**
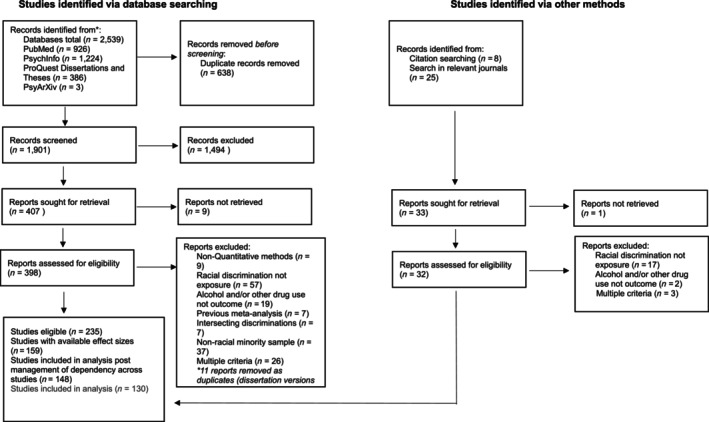
Flowchart of the study selection process.

Combinations of the following subject headings/index terms and free text terms were searched in PubMed, PsychInfo via Ovid, ProQuest for Dissertations and Theses and PsyArXiv: ‘Racism’, ‘racial discrimination’, ‘ethnic discrimination’, ‘racial trauma’, ‘racial abuse’, ‘substance use’, ‘drug use’, ‘addiction’, ‘drug abuse’, ‘alcohol use’, ‘substance abuse’, ‘substance use disorders’, ‘alcoholism’, ‘smoking’, ‘tobacco use’, ‘cannabis use’, ‘marijuana use’, ‘cannabis abuse’, ‘marijuana abuse’, ‘illicit substance use’, ‘opioid use’, ‘amphetamine use’, ‘cocaine use’ were searched. No filters were added to the search, except in ProQuest, to specify that only dissertations and theses should be returned. Searches were performed from January to July 2023 and updated in September 2024. In addition, the Journal of Psychoactive Drugs, Journal of Cultural Diversity and Ethnic Minority Psychology, Addictive Behaviours, Psychology of Addictive Behaviours, Journal of Immigrant and Minority Health, Journal of Ethnicity in Substance Abuse and Substance Use and Misuse were reviewed for additional studies that were not captured by the search strategy. These journals were selected as approximately a quarter of the studies identified via the search strategy were published in these journals. Likewise, citation searching was conducted on previous meta‐analyses and systematic reviews of racial discrimination and health outcomes. See Figure 1.

### Eligibility criteria

Inclusion criteria included: use of quantitative methodologies, studies that examined an association between racial discrimination (and known synonyms, i.e. racial harassment, racial bullying) and any type or pattern of AOD (excluding treatment outcomes and measures of craving, relapse or intentions/willingness to use) and samples, which are comprised of people who are members of minoritised racial/ethnic groups.

Measures of racial discrimination included those that capture the frequency, chronicity or count of racially discriminatory events and those that capture racial discrimination appraisal.

Exclusion criteria included: studies that include racial discrimination in a composite measure of general or intersecting discriminations, and/or studies that constitute a previous meta‐analysis/systematic review on the association between racial discrimination and AOD.

In cases where studies have used two‐stage attribution style questionnaires to measure racial discrimination (i.e. the everyday discrimination scale, experiences of discrimination scale), these were only deemed eligible if they had been attributed to race, ethnicity or nationality.

### Screening procedure

Screening was performed by two independent reviewers (E.G. and M.C.). First, titles and abstracts were screened against the eligibility criteria in Rayyan Screening Software for Systematic reviews [50]. Second, full‐text versions of studies identified as potentially eligible in the first screening phase were retrieved. Two independent reviewers screened each full‐text article against the eligibility criteria and recorded reasons for ineligibility when applicable. A third independent reviewer also determined eligibility in instances of disagreement (Figure 1).

### Coding procedure and data extraction

Two trained and independent coders (E.G. and M.C.) extracted data from full‐text articles onto piloted coding forms. Where data was missing, authors were contacted to obtain this information. Discrepancies between coders were resolved via discussion and reappraisal of the full text articles. Data was extracted into five categories: methodological data (e.g. study design, method of recruitment), participant data (e.g. sample race/ethnicity, gender and age), exposure data (e.g. measurement of exposure, timing of exposure), outcome data (e.g. measure of outcome, operationalisation of outcome) and results data (e.g. statistical models used, effect sizes, 95% CI). The effect sizes extracted included correlation coefficients, OR and β coefficients.

### Quality and certainty assessments

Assessment of study quality was performed by E.G. using the National Institute of Health study quality assessment tool for observational cohort and cross‐sectional studies [51]. Studies were assigned a quality rating of poor, fair or good based on assessment tool scores of <50%, 50% to 75% and >75%, respectively. Studies classified as poor were not excluded from analysis, because it has been suggested that there are dangers to blindly excluding poor‐rated studies from systematic reviews/meta‐analysis, as there are no clear‐cut distinctions between high‐ and low‐quality studies. Likewise, study quality assessment tools can only establish whether a study is susceptible to bias, and not whether it is biased [52]. Therefore, alternatively, a sensitivity analysis with the removal of poor rated studies was performed, in line with recommendations for meta‐analysis of observational studies [53].

Certainty assessment was performed by E.G. using the GRADE framework, where the strength of evidence was determined through appraisals of risk of bias, inconsistency, indirectness, imprecision and publication bias [54]. As the evidence included in this review was obtained from observational studies, the certainty of evidence rating for each outcome was initially low [55]. Certainty assessment can be found in Data S1.

## DATA SYNTHESIS AND STATISTICAL ANALYSIS

### Effect size metric

Correlation coefficients (*r*) were the effect size metric in this analysis. Where correlation coefficients were not reported, but other effect sizes were, these were converted to correlation coefficients using effect size converters, where possible [56, 57]. Only unadjusted effect sizes were converted because of inconsistencies in the type and number of covariates included in adjusted analyses across studies. Spearman ρ correlations were converted to approximate Pearson correlations using the equation provided by Rupinski and Dunlap [58]. For continuous AOD outcomes, Pearson's, biserial and tetrachoric correlations represented the metrics of interest, because they are statistically comparable metrics that capture underlying continuous constructs [59]. For true dichotomous outcomes, the point‐biserial correlation represented the metric of interest. In outcome domains that contained a combination of biserial, Pearson and tetrachoric coefficients, analysis was performed using raw coefficients, as Fisher's r‐z transformation is inappropriate when combining these types of correlations [59]. However, for outcome domains that only contain Pearson coefficients, the r‐z transformation was performed for analysis and back transformed for interpretation [60, 61].

### Management of dependent effect sizes

Samples represent the unit of analysis in this study and are treated as independent. Therefore, only one effect size was included per sample/sub‐sample, per AOD outcome. To ensure this, two sets of prioritisation criteria were developed to determine, which dependent effect sizes were included in the analysis. These criteria addressed dependency within and between studies and can be found in Data S2.

### Outcomes of interest

Effect sizes were categorised into discrete outcome domains if ≥4 effect sizes were available for each domain [62]. Therefore, some of the extracted effect sizes could not be included in this meta‐analysis as their corresponding AOD outcome occurred at a frequency of less than 4. Categorisation of outcomes resulted in 17 distinct AOD domains. See Table 1 for operationalisation and measurement of the outcome domains.

**TABLE 1:** Outcomes of interest.

AOD outcome	Operationalisation/definition	Example measurements
Tobacco use	Frequency of use Quantity of use Frequency × quantity of use	•CDC College Health Risk Behaviour Survey •Monitoring and Future National Survey •Bespoke scales^a^
Smoking status	Outcomes which categorised participants as ‘smokers’ or ‘non‐smokers’ via self‐identification or classification based on responses to smoking‐related questions	•US National Health Interview Survey •Sample Adult Core Questionnaire •Bespoke scales^a^
Presence–absence of tobacco use	Indication of the presence or absence of tobacco use	•National Household Survey on Drug Abuse •Monitoring the Future survey
Alcohol use	Frequency of use Quantity of use Frequency × quantity of use	•AUDIT consumption only items •CDC Youth Risk Behaviour Survey •Monitoring and Future National Survey •Daily Drinking Questionnaire •Adolescent Drinking Questionnaire •Drinking Styles Questionnaire •Youth Risk Behaviour Surveillance Scale •Timeline Follow Back •World Health Composite International Diagnostic Interview •Bespoke scales^a^
Alcohol use disorder	Indication of alcohol abuse or dependence as per DSM‐IV or alcohol use disorder as per DSM‐V	•Alcohol Use Disorder and Associated Disabilities Interview Schedule‐5 •World Mental Health Composite International Diagnostic Interview •Alcohol Use Disorder and Associated Disabilities Interview Schedule‐4 •Diagnostic Interview Schedule for Children
Binge drinking	Frequency of consuming 4/5 drinks on one occasion	•AUDIT (binge drinking item) •CDC and Prevention's behavioural risk factor surveillance system questionnaire •Timeline follow back •Youth Risk Behaviour Surveillance Scale •Bespoke scales^a^
At‐risk/hazardous alcohol use	Sum of scores or positive indication for AUDIT and CAGE or outcomes, which captured a combination of alcohol consumption, problems and dependency	•AUDIT •CAGE •Bespoke scales^a^
Alcohol problems/consequences	Frequency or number of problems/consequences related to alcohol use	•AUDIT (problem items only)/AUDIT‐P •Drinker Inventory of Consequences •Rutgers Alcohol Problem Index •Addiction severity index •Brief Michigan Alcoholism Screening test •Young adult alcohol consequences questionnaire •Bespoke scales^a^
Presence–absence of alcohol use	Indication of the presence or absence of alcohol use	•National Household Survey on Drug Abuse •Bespoke scales^a^
Cannabis use	Frequency of use Quantity of use Frequency × quantity of use	•Youth Risk Behaviour Surveillance Scale •Monitoring the Future •World Health Composite International Diagnostic Interview •Youth risk behaviour survey •Bespoke scales^a^
Presence–absence of cannabis use	Indication of the presence or absence of cannabis use	•National Household Survey on Drug Abuse •Bespoke scales^a^
Cannabis problems/consequences	Frequency or number of problems/consequences related to cannabis use	•Brief Marijuana Consequences Questionnaire •Bespoke scales^a^
At‐risk/hazardous cannabis use	Sum of scores or positive indication on CUDIT and its revised version	•CUDIT
Illicit substance use	Frequency of use Quantity of use Frequency × quantity of use Referring to substances that are prohibited by law and the use of prescription drugs without a prescription from a medical professional. Cannabis was modelled as a separate outcome to illicit substance use because of the variability in its legality status across time, countries and states	•Youth Risk Behaviour Surveillance Scale‐Illicit drug use subscale •Monitoring the Future •World Health Composite International Diagnostic Interview •Youth risk behaviour survey •The Diagnostic Interview Schedule for Children •Addiction severity index •Bespoke scales^a^
Substance use disorder	Indications of substance abuse or dependence as per DSM‐IV or substance use disorder as per DSM‐V, excluding alcohol and tobacco	•World Mental Health Composite International Diagnostic Interview •DSM‐V criteria •National Institute of Alcohol Abuse, Alcoholism, Alcohol use disorder and Associated Disability Interview Schedule •Alcohol Use Disorder and Associated Disabilities Interview Schedule‐5
Substance use problems	Frequency or number of problems/consequences related to substance use, excluding alcohol and tobacco	•Minnesota Survey of Substance Use Problems •DAST •Addiction Severity Index •Bespoke scales^a^

Abbreviations: AOD, alcohol and other drug use; AUDIT, Alcohol Use Disorder Identification Test; CAGE, Cut‐down, Annoyed, Guilty and Eye‐open questionnaire; CDC, Center of Disease Control; CUDIT, Cannabis Use Disorder Identification Test; DAST, Drug Abuse Screening Test; DSM, Diagnostic and Statistical Manual of Mental Disorders; US, United States.

^a^ Bespoke scales refer to non‐standardised or psychometrically tested measures/questionnaires.

### Statistical analysis

A series of 17 univariate meta‐analytic models, using inverse‐variance weighting, were implemented.

Random‐effects models were chosen as effect sizes were expected to vary across studies.

Heterogeneity was assessed via Cochran's Q test, where *P* < 0.05 indicates heterogeneity [63]. The I^2^ statistic was used to identify the percentage of variation in effect sizes because of heterogeneity, where a value of 75% indicates considerable heterogeneity [63, 64]. The statistical analysis was performed in R Studio using the *meta, dmetar* and *metasens* packages.

Calculating a summary effect size across all 17 outcomes was not possible because of violations of the assumption of independence. As some studies reported separate effect sizes for multiple outcomes for the same sample, combining them would result in participant duplication and dependent effects. Moreover, for studies with shared secondary samples, management of dependency was conducted at the outcome level to ensure only one effect size per sample was present in each univariate model; therefore, collating them within one model would result in dependent effects [65, 66].

### Subgroup analysis

Where possible, subgroup analysis was performed to explore heterogeneity. The moderators of interest in this analysis were race/ethnicity, age group, gender, study design and timing of exposure. Analysis of subgroups was only possible for subgroups containing ≥2 effect sizes. Our approach for identifying subgroups was data‐driven, whereby the categorisation of subgroups was informed by the studies deemed eligible for inclusion and their associated sample and methodological characteristics.

### Publication bias

Publication bias assessment for outcomes with >10 effect sizes was conducted via visual inspection of funnel plots and the Eggers test, where a significant (*P* < 0.05) result indicates plot asymmetry [67, 68]. However, for outcomes with ≤10 effect sizes, tests of funnel plot asymmetry are underpowered [69, 70]. As such, doiplots were created and inspected for asymmetry, and the Luis Furuya‐Kanamori (LFK) index was used to quantify asymmetry. In which a value that falls between −1 and 1 was deemed to be indicative of plot symmetry [71].

## RESULTS

### Study selection

A total of 1901 studies were identified via database searching and screened for eligibility at the title and abstract level, where 1494 were excluded for violating the eligibility criteria. A total of 398 were screened at the full‐text level, where 225 were deemed eligible. A further 32 records were identified via citation and journal searching and underwent full‐text screening, where 10 were considered eligible. The results were that 235 studies were eligible, 159 of which provided relevant effect sizes or data to calculate an effect size, 11 studies were removed from the analytical pool during the dependency management process across studies, and a further 18 studies were removed as their AOD outcomes occurred at a frequency of <4. Therefore, 130 studies, with 273 effect sizes, contributed to this analysis (Figure 1).

### Identification of subgroups

Subgroups were categorised into the following: age groups—youth and/or adolescents, young adults and/or adults and mixed (i.e. multiple age groups); gender—male, female and mixed (i.e. both male and female participants); study design—longitudinal and cross‐sectional; exposure timing—lifetime, past year and less than past year; and minoritised racial/ethnic groups—Black American (African and/or Afro‐Caribbean heritage and residing in the United States), Latinx (Central and Southern American heritage and residing in the United States), Asian American (Asian heritage and residing in the United States), Indigenous North American (Indigenous peoples of North America), Black Canadians (African and/or Afro‐Caribbean heritage and residing in Canada), Aboriginal Australian and Torres Strait Islander (Indigenous Australian or Torres Strait Islands heritage and residing in Australia), South Asian (South Asian heritage and residing in Hong Kong), multi‐racial/ethnic (heritage from multiple racial/ethnic groups, irrespective of country), and diverse (multiple different racial/ethnic groups not analysed separately, irrespective of country).

The diverse race/ethnicity group, the mixed gender group and the mixed age group were not considered meaningful categories for comparison and were not included in the subgroup analyses.

### Study characteristics

Characteristics of each study are displayed in Table 2. Studies were conducted/published between 1997 and 2024. Sample sizes ranged from 55 to 17 115 and 75% of studies used cross‐sectional designs, and the length of follow‐up for longitudinal studies ranged from up to 3 weeks to 13 years. A total of 109 of the studies were published in academic journals, 20 were dissertations/theses and one was a pre‐print. The majority of studies were conducted in the United States (94%), and the remainder were conducted in Canada, Hong Kong and Australia. In the US‐based studies, 56 had Black American‐only samples, 24 had Latinx‐only samples, nine had Asian American‐only samples, six had Indigenous North American‐only samples and one had a multi‐racial/ethnic only sample. In the Canada‐based studies, one had a Black Canadian‐only sample and two had Indigenous North American‐only samples. The Australia‐based study had an Aboriginal and Torres Strait Islander only sample, and the Hong Kong‐based study had a South Asian only sample. The remaining studies had a diverse sample of minoritised racial/ethnic groups that were not analysed separately or had multiple different minoritised racial/ethnic groups that were stratified for analytical purposes. A total of 70% of studies used a secondary data source, and 73% of studies had predominantly female samples and mean ages ranged from 9.5 to 49. With regards to methodological quality, 50 studies were rated as poor, 77 were rated as fair and three were rated as good. The most common methodological quality concerns among ‘poor’ rated studies were: a lack of clarity regarding the study eligibility criteria, inability to determine if the exposure preceded the outcome and insufficient information or no information regarding the reliability and validity of the outcome measurement.

**TABLE 2:** Study characteristics.

Study author and date	*n*	Design	Type	Country	Outcomes	Racial discrimination measurement	Secondary source	% female	Mean age	Race/ethnicity	Effect size	Study quality
Khazvand *et al*. 2022 [72]	501	Cross‐sectional	Journal	United States	Composite substance use	Index of Race–Related Stress‐Brief	Unknown	59.50	23.75	Diverse	0.17	Poor
Layland 2020 [73]	217	Longitudinal (2 y)	Dissertation	United States	Alcohol use problems; alcohol use; at‐risk alcohol use; binge drinking; cannabis use; tobacco use; substance use disorder	Bespoke scale^a^	Healthy Young Mens Cohort	NA	22.3	Diverse	0.21; 0.13; 0.16; 0.13; 0.16; 0.23; 0.22	Fair
Lee *et al*. 2018 [74]	465	Longitudinal (4 y)	Journal	United States	Alcohol use problems; alcohol use	Daily Life Experiences scale	Flint Adolescent study	Unknown	Unknown	Black American	0.16; 0.25	Fair
Gerrard *et al*. 2017 [75]	508	Longitudinal (9 y)	Journal	United States	Alcohol use problems; alcohol use	Schedule of Racist Events (modified)	FACHS	94	Unknown	Black American	0.07; −0.01	Fair
Tran 2016 [76]	131	Cross‐sectional	Dissertation	United States	At‐risk alcohol use	Asian American Racism‐Related Stress Inventory	NA	50.40	32.49	Asian American	0.09	Fair
Drazdowski *et al*. 2016 [77]	200	Cross‐sectional	Journal	United States	Cannabis use; illicit substance use	Racism and Life Experiences scale (daily life experiences subscale)	Unknown	53	Unknown	Diverse	0.00; 0.17	Fair
Lorenzo‐Blanco *et al*. 2015 [78]	1919	Longitudinal (2 y]	Journal	United States	Tobacco use	Unknown	Project RED	52	14.1	Latinx	0.07	Fair
Sanders‐Phillips *et al*. 2014 [79]	567	Cross‐sectional	Journal	United States	Alcohol use; cannabis use	Bespoke scale^a^	Unknown	61	15.6	Black American	0.06; 0.00	Poor
Ornelas *et al*. 2011 [80]	275	Cross‐sectional	Journal	United States	Binge drinking	Bespoke scale^a^	Men as Navigators for Health and Hombres Manteniendo Bienestar y Relaciones Saludables	0	28.4	Latinx	0.09	Fair
Copeland‐Linder *et al*. 2010 [81]	232‐268	Longitudinal (2 y)	Journal	United States	Alcohol use; cannabis use; tobacco use	Racism and Life Experiences Scale	Unknown	Unknown	46.40	Black American	0.01; 0.06; −0.07; 0.07; 0.01; 0.02	Fair
Flores *et al*. 2010 [82]	110	Longitudinal (6 months)	Journal	United States	Alcohol use; illicit substance use	Discrimination Stress Scale	NA	46	18.8	Latinx	0.35; 0.22; 0.16	Fair
Stone *et al*. 2017 [83]	4249	Cross‐sectional	Journal	United States	Alcohol use; cannabis use; tobacco use	Bespoke scale^a^	Health Behaviours in School Aged Children	51.7; 54.1; 49.7; 50.6; 50.8	Unknown	Black American; Latinx; Asian American; other ethnic minority, multi‐racial/ethnic	−0.13; 0.05; 0.02; 0.05; 0.14; −0.097; 0.03; 0.17; −0.06; −0.03; −0.097; ‐0.03; −0.5; −0.03; 0.05	Poor
Berkel *et al*. 2022 [84]	571	Longitudinal (1 y)	Journal	United States	Substance use problems	Racism and Life Experiences Scale	NA	54	Unknown	Black American	0.29	Fair
Mata‐Greve *et al*. 2018 [85]	233	Cross‐sectional	Journal	United States	At‐risk alcohol use	The Brief Perceived Ethnic Discrimination Questionnaire	NA	73	36.32	Latinx	0.23	Poor
Liu *et al*. 2022 [86]	289	Cross‐sectional	Journal	United States	At‐risk alcohol use	AAPI Hate Reporting Centre's Incident Report Questionnaire	NA	43	33.1	Asian American	−0.09	Fair
Song *et al*. 2022 [87]	602	Longitudinal (5 y)	Journal	United States	Alcohol use	Bespoke scale^a^	Unknown	54	12.92	Latinx	0.03	Fair
Su *et al*. 2022 [88]	383	Cross‐sectional	Journal	United States	Alcohol use problems	Schedule of Racist Events	Cultural Experiences and Alcohol Use study	81	20.65	Black American	0.13	Fair
Iwamoto *et al*. 2022 [89]	1432	Cross‐sectional	Journal	United States	Alcohol use problems	The Everyday Racial Discrimination Scale	Unknown	73.20	19.81	Asian American	0.22	Fair
Heads *et al*. 2020 [90]	266	Cross‐sectional	Journal	United States	At‐risk alcohol use; illicit substance use	The Scale of Ethnic Experience ‐ Perceived Discrimination subscale	Multi‐site university study of identity and culture	71.80	19.8	Black American	0.14; 0.1	Fair
Zimmerman *et al*. 2022 [91]	1333	Cross‐sectional	Journal	United States	Presence–absence of alcohol use; Presence–absence of cannabis use, Presence–absence of tobacco use	Unknown	Project on Human Development in Chicago Neighbourhoods	52.44	17.6	Diverse	0.23; 0.2; 0.17	Poor
Steele *et al*. 2022 [92]	291	Longitudinal (unknown length)	Journal	United States	Binge drinking	Schedule of Racist Events	FACHS	100	Unknown	Black American	0.07	Fair
Schick *et al*. 2021 [93]	106	Cross‐sectional	Journal	Canada	Alcohol use; alcohol use problems	Bespoke scale^a^	Unknown	50	14.6	Indigenous North American	0.3; 0.29	Fair
Crichlow *et al*. 2022 [94]	1514	Cross‐sectional	Journal	United States	Composite substance use	Bespoke scale^a^	Unknown	56.90	13.56	Black American	0.12	Poor
Su *et al*. 2021 [95]	165	Cross‐sectional	Journal	United States	Alcohol use	Daily Life Experiences scale	Unknown	75	21.56	Black American	0.09	Poor
Keum *et al*. 2021 [96]	387	Cross‐sectional	Journal	United States	At‐risk alcohol use	Perceived Online Racism Scale	NA	57	Unknown	Diverse	0.47; 0.37	Poor
Zapolski *et al*. 2021 [97]	399	Cross‐sectional	Journal	United States	At‐risk alcohol use; at‐risk cannabis use	The Racial and Ethnic Microaggression scale	NA	61.40	20.7	Black American	0.23; 0.24	Fair
Bakhtiari *et al*. 2020 [98]	121	Cross‐sectional	Journal	United States	Cannabis use	Adolescent Discrimination Distress index	Schools, Peers, and Adolescent Development Project	54	15.56	Latinx	0.02	Poor
Brown *et al*. 2021 [99]	399	Cross‐sectional	Journal	United States	Illicit substance use	Everyday Discrimination scale	NA	26.80	34	Diverse	0.4	Poor
Nalven *et al*. 2021 [100]	598	Cross‐sectional	Journal	United States	Binge drinking	Unknown	National Epidemiologic Survey on Alcohol and Related Conditions‐III	54.40	40.28	Diverse	0.095	Poor
Clifton *et al*. 2021 [101]	147	Cross‐sectional	Journal	United States	Composite substance use	Daily life Experiences Scale	NA	81.70	23.16	Black American	0.22	Poor
Marks *et al*. 2021 [102]	196	Cross‐sectional	Journal	United States	Alcohol use; alcohol use problems	Inventory of Microaggressions Against Black Individuals	NA	Unknown	Unknown	Black American	0.2; 0.01	Fair
Motley *et al*. 2021 [103]	300	Cross‐sectional	Dissertation	United States	Alcohol use problems; alcohol use; illicit substance use; substance use problems	Classes of Racism Frequency of Racial Experiences scale	NA	50.70	19	Black American	0.014; 0.256; 0.15; 0.131	Fair
Waldron *et al*. 2021 [104]	245	Longitudinal (1 y)	Journal	United States	Alcohol use; alcohol use problems	Schedule of Racist Events (modified)	NA	73.10	Unknown	Latinx	0.23; 0.09	Fair
Kogan *et al*. 2020 [105]	505	Longitudinal (3 y)	Journal	United States	Binge drinking; cannabis use	Schedule of Racist Events	African American Mens Project	0	20.26	Black American	0.1; 0.14	Fair
Glass *et al*. 2020 [106]	17 115	Cross‐sectional	Journal	United States	Alcohol use disorder	Experiences of Discrimination scale (modified)	National Epidemiologic Survey On Alcohol and Related Conditions III	52.60	Unknown	Diverse	0.11	Fair
Lui 2020 [107]	988	Cross‐sectional	Journal	United States	Alcohol use problems; alcohol use	Everyday Discrimination scale	NA	42; 60.4; 64.7	22.42; 22.69; 23.02	Black American; Asian American; Latinx	−0.16; 0.23; 0.08; 0.05; 0.08; −0.06	Poor
Meca *et al*. 2020 [108]	1101	Longitudinal (3 y)	Journal	United States	Alcohol use	Unknown	Project RED	51.20	13.99	Latinx	0.14	Fair
Jelsma *et al*. 2019 [109]	610	Longitudinal (3 y)	Journal	United States	Alcohol use; cannabis use	Bespoke scale^a^	Maryland Adolescent Development in Context Study	49	Unknown	Black American	0.17; 0.16	Fair
Su *et al*. 2020 [110]	383	Cross‐sectional	Journal	United States	Alcohol use	Schedule of Racist Events	Cultural Experiences and Alcohol Use study	81	20.65	Black American	0.07	Fair
Hicks *et al*. 2018 [111]	505	Longitudinal (1.5 y)	Journal	United States	Tobacco use	Schedule for Racist Events (modified)	African American Men's Health Project	0	20.26	Black American	0.19	Fair
Nieri *et al*. 2022 [112]	259	Longitudinal (unknown length)	Journal	United States	Alcohol use; binge drinking; cannabis use	Unknown	NA	61	15	Diverse	−0.088; −0.033; 0.004	Poor
Piña‐Watson *et al*. 2019 [113]	796	Cross‐sectional	Journal	United States	At‐risk alcohol use	Perceived racism scale for Latinxs ‐ Frequency of exposure to racism subscale	NA	66.30	19.45	Latinx	0	Fair
Desalu *et al*. 2017 [42]	251	Cross‐sectional	Journal	United States	Alcohol use problems; binge drinking	The Perceived Ethnic Discrimination Questionnaire	NA	66	20	Black American	0.3; −0.01	Fair
Le *et al*. 2019 [114]	311	Longitudinal (1 y)	Journal	United States	Alcohol use; alcohol use problems	Everyday Discrimination scale	Unknown	55	18.1	Asian American	0.15; 0.01	Fair
Dickerson *et al*. 2019 [43]	182	Cross‐sectional	Journal	United States	Alcohol use problems; alcohol use; binge drinking; cannabis use; tobacco use; cannabis use problems	Microaggressions Distress Scale	NA	50	15.6	Indigenous North American	0.35; 0.13	Fair
Franco *et al*. 2019 [115]	466	Cross‐sectional	Journal	United States	Composite substance use	Multiracial Challenge and Resilience scale (General discrimination subscale)	NA	66.20	29.7	Multi‐racial/ethnic	0.1	Poor
Zapolski *et al*. 2019 [116]	612	Cross‐sectional	Journal	United States	Composite substance use	Bespoke scale^a^	Unknown	58.40	Unknown	Black American	0.15	Poor
Zapolski *et al*. 2018 [117]	388	Cross‐sectional	Journal	United States	Alcohol use	Schedule of Racist Events	NA	62.40	20.6	Black American	0.56	Fair
Gibbons *et al*. 2018 [118]	889	Longitudinal (13 y)	Journal	United States	Smoking status; tobacco use	Schedule of Racist Events	FACHS	54	15.5	Black American	0.1; 0.12	Fair
Lee et al. 2018 [119]	681	Longitudinal (3 y)	Journal	United States	At‐risk alcohol use	Daily Life Experiences scale	Unknown	51	Unknown	Black American	0.1	Fair
Metzger *et al*. 2018 [120]	235	Cross‐sectional	Journal	United States	Alcohol use; binge drinking	Daily Life Experiences scale	Activties and Behaviours in College study	73.60	20.56	Black American	0.16; 0.21	Poor
Pittman *et al*. 2018 [121]	469	Cross‐sectional	Journal	United States	At‐risk alcohol use	Index of Race‐Related Stress (brief version)	Unknown	100	20.24	Black American	0.18	Poor
Ornelas *et al*. 2015 [122]	5313	Cross‐sectional	Journal	United States	Binge drinking	Brief Perceived Ethnic Discrimination Questionnaire (community version)	Hispanic Community Health study/Study of Latinos ‐ Sociocultural ancillary study	55	42	Latinx	0.1	Fair
Demianczyk 2015 [123]	418	Cross‐sectional	Dissertation	United States	Alcohol use problems	The Racial and Ethnic Microaggression scale	NA	Unknown	Unknown	Asian American; Black American; Latinx; multi‐racial/ethnic	0.44; 0.35; 0.38; 0.3	Poor
Zapolski *et al*. 2016 [124]	1521	Cross‐sectional	Journal	United States	Composite substance use	Bespoke scale^a^	Unknown	56.30	Unknown	Black American	0.07	Poor
Thompson *et al*. 2016 [125]	144	Cross‐sectional	Journal	United States	Alcohol use; at‐risk alcohol use, smoking status	Experiences of Discrimination scale	The Black LIFE	52	44.6	Black American	0.0325; 0.229; 0.58	Poor
Greenfield 2015 [126]	347	Cross‐sectional	Dissertation	United States	Alcohol use; binge drinking; illicit substance use	The Microaggressions scale	NA	61.50	28.45	Indigenous North American	0.022; 0.014; 0.134	Fair
Armenta *et al*. 2015 [127]	674	Longitudinal (7 y)	Journal	United States; Canada	Alcohol use disorder; presence–absence of alcohol use	Schedule of Racist Events	Unknown	Unknown	Unknown	Indigenous North American	0.15; 0.07	Good
Tse *et al*. 2014 [128]	202	Cross‐sectional	Journal	Hong Kong	At‐risk alcohol use	Unknown	NA	0	Unknown	South Asian	0.03	Poor
Rodriguez‐Seijas *et al*. 2015 [129]	5191	Cross‐sectional	Journal	United States	Alcohol use disorder; substance use disorder	Bespoke scale^a^	National Survey of American Life	Unknown	Unknown	Black American	0.23; 0.22	Poor
Brondolo *et al*. 2015 [130]	518	Longitudinal (up to 3 weeks)	Journal	United States	Smoking status	Perceived Ethnic Discrimination Questionnaire (community version)	Unknown	Unknown	Unknown	Black American	0.19; 0.03	Fair
Cano *et al*. 2015 [131]	129	Cross‐sectional	Journal	United States	At‐risk alcohol use	Environmental scale from the Social attitudes, Familial, and Environmental Acculturation scale	NA	70	19.41	Latinx	0.23	Poor
Cheng *et al*. 2015 [132]	203	Longitudinal (1 y)	Journal	United States	At‐risk alcohol use	General Ethnic Discrimination scale	NA	59.10	24.06	Latinx	0.18	Fair
Spence *et al*. 2014 [133]	340	Cross‐sectional	Journal	Canada	Presence–absence of cannabis use	Measure of Indigenous Racism Experience Interpersonal racism scale	Researching Health in Ontario Communities	54.90	41	Indigenous North American	0	Fair
Kapadia 2013 [134]	13 914	Cross‐sectional	Dissertation	United States	Alcohol use disorder	Experiences of Discrimination scale	National Epidemiologic survey on Alcohol and related conditions II	61	Unknown	Diverse	0.16; 0.14	Fair
Hurd *et al*. 2014 [26]	681	Longitudinal (3 y)	Journal	United States	Alcohol use; tobacco use	Unknown	Unknown	Unknown	Unknown	Black American	0.21; 0.02	Fair
Otiniano Verissimo *et al*. 2014 [135]	2312	Cross‐sectional	Journal	United States	Alcohol use; alcohol use disorder; illicit substance use; substance use disorder	Everyday Discrimination scale	NLAAS	55	Unknown	Latinx	0.11; 0.13; 0.4; 0.23; 0.24; 0.22; 0.5; 0.25	Fair
Latzman *et al*. 2013 [136]	336	Cross‐sectional	Journal	United States	Alcohol use problems	Unknown	NA	70.50	20.4	Diverse	0.56	Poor
Borrell *et al*. 2012 [137]	1169	Longitudinal (13 y)	Journal	United States	Presence–absence of alcohol use	Bespoke scale^a^	CARDIA	Unknown	Unknown	Black American	0.21	Fair
Thoma *et al*. 2013 [138]	276	Cross‐sectional	Journal	United States	Alcohol use; binge drinking; cannabis use; tobacco use	Schedule of Racist Events	Diverse Adolescents Sexual Health study	33	17.45	Black American	−0.02; 0.15; 0.22; 0.1	Poor
Horton *et al*. 2011 [139]	573	Cross‐sectional	Journal	United States	Tobacco use	General Ethnic Discrimination scale	Unknown	47	Unknown	Black American; Latinx	0.17; 0.03	Poor
Gibbons *et al*. 2013 [140]	889	Longitudinal (8 y)	Journal	United States	Composite substance use	Schedule of Racist Events	FACHS	54	18.5	Black American	0.1	Good
Park 2010 [141]	1628	Cross‐sectional	Dissertation	United States	Alcohol use	Bespoke scale^a^	NLAAS	52.50	41.6; 41.9; 43	Asian American	0.05; −0.03; 0.23	Poor
Respress 2010 [142]	514	Cross‐sectional	Dissertation	United States	Alcohol use; binge drinking; cannabis use; presence–absence of cannabis use	Bespoke scale^a^	The National Longitudinal Study of Adolescent Health	52.50	Unknown	Black American	0.042; 0.082; 0.196; 0.16	Poor
Krieger *et al*. 2011 [143]	504	Cross‐sectional	Journal	United States	Smoking status	Everyday Discrimination scale	My body, My story	69.20	48.6	Black American	0.01	Fair
Kam *et al*. 2011 [144]	728	Longitudinal (2 y)	Journal	United States	Composite substance use	Bespoke scale^a^	Unknown	54	12.3	Latinx	0.11	Fair
Galliher *et al*. 2010 [145]	133	Cross‐sectional	Journal	United States	Composite substance use; substance use problems	Unknown	NA	Unknown	Unknown	Indigenous North American	0.371; −0.229	Fair
Borrell *et al*. 2010 [44]	2266	Cross‐sectional	Journal	United States	Smoking status	Detroit Area Study Discrimination Questionnaire	Multi‐ethnic study of Atherosclerosis	51.4; 51.9	Unknown	Asian American; Latinx	0.1; 0.14	Poor
Kwate *et al*. 2009 [146]	139	Cross‐sectional	Journal	United States	Binge drinking; at‐risk alcohol use	Daily Life Experiences scale (race and bother scales)	Unknown	100	34	Black American	0.071; −0.097	Fair
Yoo *et al*. 2009 [147]	271	Cross‐sectional	Journal	United States	Presence–absence of alcohol use	Unknown	Asian Pacific Arizona Initiative survey	52.20	43.6	Asian American	0.05	Poor
Semino 2008 [148]	110	Cross‐sectional	Dissertation	United States	At‐risk alcohol use	Schedule of Racist Events	NA	100	32.6	Black American	0.13	Fair
Kulis *et al*. 2009 [149]	1374	Cross‐sectional	Dissertation	United States	Alcohol use; cannabis use; illicit substance use; tobacco use	Bespoke scale^a^	Unknown	51.20	10.36	Latinx	0.14; 0.165; 0.119; 0.154	Poor
Gibbons *et al*. 2007 [150]	606	Longitudinal (5 y)	Journal	United States	Cannabis use; illicit substance use	Schedule of Racist Events	FACHS	Unknown	10.5	Black American	0.15; 0.08	Fair
Kwate *et al*. 2003 [151]	33–34	Longitudinal (unknown length)	Journal	United States	Alcohol use; tobacco use	Schedule of Racist Events	Unknown	100	44.4	Black American	0.23; 0.13	Poor
Guthrie *et al*. 2002 [152]	178	Cross‐sectional	Journal	United States	Presence–absence of tobacco use	Everyday Discrimination scale	Female Adolescent Substance Experience study	100	15.45	Black American	0.35	Poor
Lui *et al*. 2020 [153]	740	Cross‐sectional	Pre‐print	United States	At‐risk alcohol use	Everyday Discrimination scale	NA	53	Unknown	Asian American; Black American	0.1; 0.24; 0.18; 0.12	Poor
Keum *et al*. 2023 [154]	407	Cross‐sectional	Journal	United States	At‐risk alcohol use	The Perceived Online Racism scale	NA	57	34.12	Diverse	0.45	Fair
Cave *et al*. 2019 [155]	424–443	Longitudinal (5 y)	Journal	Australia	Presence–absence of alcohol use; Presence–absence of tobacco use	Bespoke scale^a^	Footprints in time: The Longitudinal study of Indigenous Children	49.60	Unknown	Aboriginal Australian and Torres Strait Islanders	0.088; 0.38	Fair
Yen *et al*. 1999 [156]	716	Cross‐sectional	Journal	United States	Alcohol use problems; at‐risk alcohol use	Bespoke scale^a^	1993–1995 San Francisco Muni Health and Safety study	Unknown	Unknown	Diverse	0.07; 0.19	Poor
Greenfield *et al*. 2021 [157]	347	Cross‐sectional	Journal	United States	Tobacco use	Experiences of Discrimination scale	NA	65.60	28.45	Indigenous North American	0.26	Poor
Assari *et al*. 2019 [158]	595	Longitudinal (13 y)	Journal	United States	Cannabis use	Daily Life Experiences scale	Flint Adolescent Study	53	14.8; 14.9	Black American	−0.04; 0.18	Fair
Tao *et al*. 2022 [159]	450	Cross‐sectional	Journal	United States	Composite substance use	Ethnic Racial Discrimination index	Unknown	52.40	20.84	Diverse	0.26	Fair
Walsh *et al*. 2021 [160]	139	Cross‐sectional	Journal	United States	At‐risk alcohol use; substance use problems	Experiences of Discrimination scale	Unknown	100	26.78	Diverse	0.03; 0.1	Fair
Currie *et al*. 2021 [161]	210	Cross‐sectional	Journal	Canada	Composite substance use	Everyday Discrimination scale	Unknown	47.10	Unknown	Diverse	0.34	Poor
Chae *et al*. 2008 [162]	2073	Cross‐sectional	Journal	United States	Alcohol use disorder	Unknown	NLAAS	52.40	41	Asian American	0.08	Fair
Keum *et al*. 2022 [163]	322	Cross‐sectional	Journal	United States	At‐risk alcohol use	Perceived Online Racism Scale	NA	57	23.3	Diverse	0.44	Fair
Otiniano Verissimo *et al*. 2014 [46]	6294	Cross‐sectional	Journal	United States	Substance use disorder	Experiences of Discrimination scale	National Epidemiological Survey on Alcohol and Related conditions II	49.18	43.82	Latinx	0.16	Fair
Squires *et al*. 2017 [164]	203	Cross‐sectional	Journal	United States	Alcohol use; smoking status	Unknown	Unknown	32	44.1	Black American	0.098; 0.15	Fair
Martin *et al*. 2019 [165]	674	Longitudinal (5 y)	Journal	United States	Composite substance use	Unknown	NA	50	Unknown	Latinx	0.13	Good
Buckner *et al*. 2022 [166]	347	Cross‐sectional	Journal	United States	Alcohol use; alcohol use problems	Perceived Ethnic Discrimination questionnaire	Unknown	Unknown	21.4	Latinx	0.15; −0.04	Fair
Buckner *et al*. 2021 [167]	160	Cross‐sectional	Journal	United States	At‐risk alcohol use	Perceived Ethnic Discrimination questionnaire	Unknown	82.90	21.7	Black American	0.27	Fair
Zaso *et al*. 2022 [168]	241	Cross‐sectional	Journal	United States	Alcohol use	Perceived Ethnic Discrimination questionnaire	Unknown	63; 72	20.03; 20.06	Black American	0.2; −0.01	Fair
Call 2001 [169]	97	Cross‐sectional	Dissertation	United States	Alcohol use; alcohol use problems; illicit substance use; substance use problems	Index of Race‐Related Stress	NA	100	33.9	Black American	0.174; 0.073; 0.216; 0.084	Fair
Woodson 2021 [170]	1146	Cross‐sectional	Dissertation	United States	Composite substance use; presence–absence of alcohol use; presence–absence of cannabis use; presence–absence of tobacco use	Everyday Discrimination scale	National Survey of American Life Adolescent supplement	50	15	Black American	0.21; 0.19; 0.14; 0.16	Poor
Breeden 2004 [171]	599	Cross‐sectional	Dissertation	United States	Cannabis use; illicit substance use	Bespoke scale^a^	Woodlawn project	51.80	Unknown	Black American	0.34; 0.37	Poor
Swann *et al*. 2020 [172]	352	Cross‐sectional	Journal	United States	At‐risk alcohol use; at‐risk cannabis use	Brief Perceived Ethnic Discrimination questionnaire (community version)	Female‐assigned at birth sexual and gender minorities	100	20.27	Diverse	0.17; 0.17; 0.26	Fair
Lee *et al*. 2014 [173]	136	Cross‐sectional	Journal	United States	Composite substance use	Bespoke scale^a^	Korean Adoption Project	54	15.16	Asian American	0.19	Poor
Greene 1997 [174]	189	Cross‐sectional	Dissertation	United States	Composite substance use	Bespoke scale^a^	NA	49.70	Unknown	Latinx	−0.27	Poor
Pittman *et al*. 2017 [175]	649	Cross‐sectional	Journal	United States	At‐risk alcohol use	Index of Race‐Related Stress (brief version)	Unknown	74.70	20.42	Black American	0.2	Fair
Whitbeck *et al*. 2002 [176]	195	Cross‐sectional	Journal	United States	Alcohol use problems; composite substance use	Unknown	NA	46	12.1	Indigenous North American	0.18; 0.29	Poor
Pro *et al*. 2017 [177]	322	Cross‐sectional	Journal	United States	Cannabis use	Racial and Ethnic Microaggression scale	NA	66.40	22.6	Diverse	0.08	Poor
Somerville 2024 [178]	199	Cross‐sectional	Dissertation	United States	Binge drinking; cannabis use; illicit substance use	Index of Race‐Related Stress	Unknown	100	Unknown	Black American	0.14; 0.2; 0.11	Fair
Arreola 2024 [179]	115	Cross‐sectional	Dissertation	United States	At‐risk alcohol use	Brief Perceived Ethnic Discrimination questionnaire (community version)	NA	81.70	Unknown	Latinx	0.28	Fair
Jones 2024 [180]	108	Cross‐sectional	Dissertation	United States	Alcohol use problems; alcohol use; alcohol use disorder; at‐risk alcohol use	Perceived Online Racism scale	NA	52.80	21.7	Black American	0.279; 0.036; 0.257; 0.231	Fair
McDowell 2023 [181]	126	Cross‐sectional	Dissertation	United States	At‐risk alcohol use; at‐risk cannabis use	Schedule of Racist Events	NA	39	23.6	Black American	0.43; 0.16	Fair
Dean 2019 [182]	152	Longitudinal (up to 2 months)	Dissertation	United States	Composite substance use	Schedule of Racist Events	NA	84.20	19.4	Black American	0.11	Fair
Centeno *et al*. 2023 [183]	703	Cross‐sectional	Journal	United States	Illicit substance use	Adult and Peer Discrimination scale	Unknown	47.60	16	Latinx	0.12	Poor
Dyar *et al*. 2023 [184]	304	Longitudinal (up to 44 days)	Journal	United States	Alcohol use problems; cannabis use problems; alcohol use; cannabis use	Experiences of Discrimination scale	Unknown	Unknown	Unknown	Black American; Latinx; other ethnic minority	0.11; −0.01; 0.07; 0.26; −0.08; 0.03; 0.07; 0.03; 0.13; −0.02; 0.09; 0.05	Fair
Mora 2023 [185]	55	Cross‐sectional	Dissertation	United States	Composite substance use	General Ethnic Discrimination scale	NA	57	Unknown	Latinx	0.35	Poor
Moreno *et al*. 2024 [186]	164	Cross‐sectional	Journal	United States	Tobacco use	Experiences of Discrimination scale (modified)	Spit for Science study	81.10	19.9	Diverse	0.27	Poor
Assari 2023 [187]	2514	Longitudinal (Up to 36 months)	Journal	United States	Presence–absence of cannabis use; Presence–absence of tobacco use	Bespoke scale^a^	Adolescent Brain Cognitive Development Study	49.50	9.5	Black American	0.083; 0.028	Fair
Buckner *et al*. 2024 [188]	164	Cross‐sectional	Journal	United States	Alcohol use problems; alcohol use	Perceived Ethnic Discrimination questionnaire	Unknown	82.90	21.7	Black American	0.27; 0.17	Fair
Zapolski and Depperman 2023 [189]	390	Cross‐sectional	Journal	United States	At‐risk alcohol use; at‐risk cannabis use	Schedule of Racist Events	NA	62	20.6	Black American	0.67; 0.24	Fair
Cénat *et al*. 2023 [190]	860	Cross‐sectional	Journal	Canada	Composite substance use	Everyday Discrimination scale	Black Community Mental Health Project	75.10	25	Black Canadian	0.348	Fair
Schick *et al*. 2023 [191]	52; 1743	Cross‐sectional	Journal	United States; Canada	Alcohol use	Perceived Discrimination scale	Our Youth, Our Future	50; 45.2	15.4	Indigenous North American	0.31; 0.14	Fair
Espinosa *et al*. 2023 [192]	7037	Cross‐sectional	Journal	United States	Substance use disorder	Experiences of Discrimination scale	National Epidemiological survey on alcohol and related conditions wave 3	56.10	39.9	Latinx	0.13	Fair
Macias Burgos *et al*. 2024 [193]	426	Cross‐sectional	Journal	United States	At‐risk alcohol use	Perceived Discrimination scale	Community Health Research and Implementation Science Data	65.50	40	Latinx	0.41	Fair
Nie 2024 [194]	356	Cross‐sectional	Journal	United States	Tobacco use	Bespoke scale^a^	NA	49.90	48.3	Asian American	0.13	Poor
Barry *et al*. 2023 [195]	510	Cross‐sectional	Journal	United States	Alcohol use; cannabis use; illicit substance use	Bespoke scale^a^	NA	52	Unknown	Indigenous North American	0.03; 0.07; 0.0	Poor
Morris *et al*. 2023 [196]	152	Cross‐sectional	Journal	United States	Alcohol use; tobacco use; cannabis use	Daily Life Experiences and Racism scale	Unknown	74.50	21.5	Black American	0.018; −0.045; 0.024	Poor

*Note*: Multiple values within one column refer to values for multiple subgroups within a study or multiple outcomes per study.

Abbreviations: AAPI, Asian American and Pacific Islander; CARDIA, Coronary Artery Risk Development in Young Adults Study; FACHS, Family and Community Health Study; NA, Not applicable; Unknown, data not available; NLAAS, National Latino and Asian American Study.

^a^ Bespoke scale refers to non‐standardised or psychometrically tested measures/questionnaires.

### Summary effect sizes

The summary effect sizes, 95% CI, *P*‐values, heterogeneity indices and prediction intervals for each AOD outcome are presented in Table 3. The data supporting these results can be found in Data S3.

**TABLE 3:** Results of main effect analyses.

AOD outcome domain	*n*	*m*	*k*	*r*	95% CI LL	95% CI UL	*P*	Q	PI LL	PI UL	I^2^ (%)
Tobacco use	13 256	17	23	0.07	0.002	0.14	0.04	250.34	−0.26	0.4	91
Alcohol use	23 623	41	55	0.09	0.06	0.13	<0.001	393.82	−0.13	0.32	86
Cannabis use	12 630	20	28	0.08	0.04	0.12	<0.001	170.46	−0.12	0.29	84.20
Illicit substance use	8022	14	15	0.18	0.12	0.24	<0.001	97.42	−0.05	0.41	85.60
Binge drinking	9601	15	15	0.09	0.07	0.11	<0.001	20.12	0.07	0.11	30.40
At‐risk/hazardous alcohol use	9445	29	33	0.24	0.17	0.3	<0.001	614.2	−0.12	0.59	94.80
Alcohol use problems/consequences	8127	23	30	0.2	0.15	0.25	<0.001	184.56	−0.06	0.47	84.30
Substance use problems/consequences	1240	5	5	0.08	−0.09	0.25	0.33	32.25	−0.5	0.62	87.60
Alcohol use disorder	41 387	7	9	0.19	0.13	0.26	<0.001	177.67	−0.05	0.43	96
Substance use disorder	21 051	5	6	0.25	0.14	0.36	<0.001	218.45	−0.15	0.64	97.70
Composite substance use	10 578	19	19	0.17	0.11	0.23	<0.001	134.6	−0.1	0.45	86.60
Smoking status	4795	7	9	0.15	0.04	0.26	0.009	80.39	−0.26	0.56	90.00
Presence–absence of alcohol use	5017	6	6	0.15	0.09	0.21	<0.0001	21.61	−0.06	0.36	76.90
Presence–absence of tobacco use	5614	5	5	0.21	0.08	0.34	0.001	78.6	−0.28	0.7	94.90
Presence–absence of cannabis use	6174	7	7	0.13	0.08	0.18	<0.001	19.68	−0.01	0.27	69.50
At‐risk/hazardous cannabis use	462	4	4	0.24	0.18	0.29	<0.001	1.01	0.12	0.35	0
Cannabis use problems/consequences	1267	2	4	0.09	0.001	0.18	0.05	2.1	−0.11	0.29	0

Abbreviations: AOD, alcohol and other drug use; CI LL, confidence interval lower limit; CI UL, confidence interval upper limit; k, number of effect sizes; m, number of studies; *n*, sample size; PI LL, prediction interval lower limit; PI UL, prediction interval upper limit.

Positive associations between racial discrimination and 16 AOD outcomes were identified. The median effect size across outcomes was 0.15. The strongest associations were observed for at‐risk/hazardous alcohol use (*r* = 0.24, 95% CI = 0.17–0.3, I^2^ = 94.8%, m = 29, *n* = 9445), at‐risk/hazardous cannabis use (*r* = 0.24, 95% CI = 0.18–0.29, I^2^ = 0%, m = 4, *n* = 462) and substance use disorder (*r* = 0.25, 95% CI = 0.14–0.36, I^2^ = 97.7%, m = 5, *n* = 21 051). The weakest association was observed for tobacco use (r = 0.07, 95% CI = 0.002–0.14, I^2^ = 91%, m = 17, *n* = 13 256). See Data S4 for forest plots. Results of the sensitivity analysis with only fair/good‐rated studies can be found in Data S5.

### Differences across AOD outcome domains

Based on non‐overlapping 95% CI, racial discrimination has a stronger association with at‐risk/hazardous drinking, alcohol use problems/consequences and alcohol use disorder (AUD) than with alcohol use. Similarly, at‐risk/hazardous cannabis use and substance use disorder had stronger associations with racial discrimination than with cannabis use. However, illicit substance use has a stronger association with racial discrimination than with cannabis use. AOD outcomes with overlapping 95% CI, however, do not necessarily indicate any differences. Although for AOD outcomes whose 95% CI contain the mean correlation coefficient for another AOD outcome, equivalent effects can be inferred [197]. As such, no differences were observed between tobacco use and alcohol use, cannabis use and smoking status. No differences were found between alcohol use, cannabis use and binge drinking. Furthermore, no differences were identified between cannabis use and cannabis problems, or substance use disorder and AUD.

### Publication bias

Across tobacco use, alcohol use, cannabis use, illicit substance use and alcohol use problems/consequences outcomes, funnel plots did not indicate asymmetry, which was supported by non‐significant Eggers tests (*P* = 0.06–0.96). Correspondingly, doiplots and the LFK index across these domains did not suggest plot asymmetry (LFKs = −0.77 to 0.6). However, for the binge drinking domain, minor asymmetry was suspected in the funnel plot, yet the Eggers test was non‐significant (*t* = −0.88, *P* = 0.4) and doiplots and LFK index indicated minor plot asymmetry (LFK = −1.36). Similarly, a non‐significant Eggers test was observed (*t* = 0.54, *P* = 0.59) for the composite substance use domain with minor plot asymmetry in doiplots and LFK index (1.53). Contrastingly, for at‐risk/hazardous alcohol use, the Eggers test indicated plot asymmetry (t = −2.92, *P* = 0.007), while doiplot and LFK index did not. Moreover, for cannabis use problems/consequences and at‐risk/hazardous cannabis use, doiplots and LFK index indicated minor plot asymmetry (LFK = −1.35, 1.92). For the substance use problems/consequences, AUD, substance use disorder, presence–absence of alcohol use, presence–absence of tobacco use domains, major plot asymmetry was detected via dioplot and LFK index (LFKs = −4.16, 3.24). Doiplots and LFK index for smoking status and presence–absence of cannabis use did not indicate plot asymmetry (LFK = 0.84, −0.35).

### Moderation analysis

Table 4 provides effect sizes, 95% CI, *P*‐values and I^2^ values for subgroup analyses.

**TABLE 4:** Summary of subgroup analyses.

AOD domain	Moderator	*k*	r	95% CI LL	95% CI UL	I^2^ (%)	*P* (subgroup differences)
Tobacco use	Study design						0.45
	Longitudinal	9	**0.1**	0.05	0.14	55.60	
	Cross‐sectional	14	0.05	−0.05	0.16	94	
	Race/ethnicity						**<0.001**
	Black American	11	**0.06**	0.01	0.12	81.70	
	Latinx	4	0.06	−0.023	0.14	89.20	
	Indigenous North American	2	**0.27**	0.2	0.35	0	
	Asian American	2	−0.18	−0.8	0.43	99	
	Gender						0.22
	Male	3	**0.14**	0.02	0.27	72.50	
	Female	2	0.03	−0.09	0.15	0	
	Age group						0.1
	Youth and adolescents	11	0.06	−0.11	0.12	94.60	
	Young adult and adults	5	**0.13**	0.04	0.22	76.20	
	Exposure timing						**0.05**
	Lifetime	3	**0.18**	0.06	0.29	62.80	
	Past year	4	0.09	−0.05	0.23	80.60	
	Less than past year	7	−0.06	−0.21	0.1	94.1	
Alcohol use	Study design						0.77
	Longitudinal	17	**0.1**	0.04	0.16	75.50	
	Cross‐sectional	38	**0.09**	0.05	0.13	88.70	
	Race/ethnicity						0.72
	Black American	27	**0.11**	0.05	0.17	91.40	
	Latinx	11	**0.08**	0.03	0.14	73	
	Asian American	6	0.06	−0.01	0.14	77.10	
	Indigenous North American	6	**0.12**	0.03	0.21	71.50	
	Gender						0.5
	Male	3	**0.09**	0.04	0.15	18.10	
	Female	4	**0.12**	0.07	0.17	0	
	Age group						0.25
	Youth and adolescents	20	**0.08**	0.04	0.13	83.60	
	Young adult and adults	13	**0.15**	0.05	0.13	92.50	
	Exposure timing						0.09
	Lifetime	6	0.06	−0.01	0.12	32	
	Past year	12	**0.16**	0.07	0.25	93.10	
	Less than past year	14	0.04	−0.02	0.09	75.6	
Cannabis use	Study design						0.95
	Longitudinal	11	**0.08**	0.03	0.14	58.10	
	Cross‐sectional	17	**0.08**	0.02	0.14	89.00	
	Race/ethnicity						0.78
	Black American	15	**0.1**	0.04	0.17	89.50	
	Latinx	4	0.08	−0.01	0.16	80.80	
	Indigenous North American	2	0.07	−0.001	0.15	0.00	
	Gender						0.9
	Male	3	0.09	−0.06	0.23	81	
	Female	3	0.07	−0.06	0.21	73.00	
	Age group						0.1
	Youth and adolescents	16	**0.05**	0.006	0.1	83.60	
	Young adult and adults	5	**0.16**	0.04	0.28	81	
	Exposure timing						0.52
	Lifetime	2	0.17	−0.16	0.5	97.30	
	Past year	6	0.08	−0.01	0.17	67.30	
	Less than past year	11	0.03	−0.02	0.09	72.3	
Illicit substance use	Study design						0.44
	Longitudinal	2	0.13	−0.003	0.26	49.30	
	Cross‐sectional	13	**0.18**	0.12	0.25	86.60	
	Race/ethnicity						0.28
	Black American	6	**0.17**	0.07	0.27	86.50	
	Latinx	5	**0.18**	0.12	0.24	72.40	
	Indigenous North American	2	0.06	−0.07	0.2	73.50	
	Age group						0.13
	Young adult and adults	2	**0.26**	0.05	0.48	90.80	
	Youth and adolescents	5	**0.10**	0.05	0.14	49.10	
	Exposure timing						**0.03**
	Lifetime	2	**0.32**	0.18	0.46	54.80	
	Past year	3	**0.15**	0.08	0.21	0	
Binge drinking	Study design						0.46
	Longitudinal	4	**0.07**	0.009	0.13	25.10	
	Cross‐sectional	11	**0.1**	0.07	0.12	35.50	
	Race/ethnicity						0.84
	Black American	8	**0.09**	0.04	0.14	45.60	
	Latinx	2	**0.1**	0.07	0.13	0	
	Indigenous North American	2	0.07	−0.05	0.19	48.40	
	Gender						0.42
	Male	3	**0.1**	0.04	0.17	0	
	Female	3	0.05	−0.08	0.17	58.40	
	Age group						0.23
	Young adult and adults	2	**0.15**	0.04	0.25	51.70	
	Youth and adolescents	3	0.06	−0.03	0.15	45.80	
At‐risk/hazardous alcohol use	Study design						**0.01**
	Longitudinal	3	**0.13**	0.07	0.19	0	
	Cross‐sectional	30	**0.24**	0.18	0.31	95.00	
	Race/ethnicity						0.06
	Black American	14	**0.27**	0.16	0.37	96.20	
	Latinx	6	**0.22**	0.1	0.34	91.70	
	Asian American	4	0.07	−0.05	0.2	74.50	
	Gender						0.1
	Male	5	**0.19**	0.04	0.35	84.70	
	Female	7	**0.19**	0.12	0.26	57.10	
	Exposure timing						0.94
	Lifetime	6	**0.23**	0.12	0.34	86.10	
	Past year	3	0.26	−0.15	0.67	99	
	Less than past year	6	**0.26**	0.09	0.44	95.80	
Alcohol use problems/consequences	Study design						**0.03**
	Longitudinal	8	**0.14**	0.08	0.19	24.90	
	Cross‐sectional	22	**0.23**	0.16	0.29	86.90	
	Race/ethnicity						0.23
	Black American	12	**0.16**	0.08	0.23	73	
	Latinx	5	**0.16**	0.05	0.27	61.30	
	Asian American	4	**0.25**	0.14	0.36	73.20	
	Indigenous North American	3	**0.28**	0.17	0.38	38.80	
	Age group						**0.04**
	Young adult and adults	11	**0.15**	0.1	0.2	49.70	
	Youth and adolescents	3	**0.28**	0.17	0.38	38.80	
	Exposure timing						0.13
	Lifetime	3	**0.08**	0.02	0.13	0	
	Past year	4	**0.16**	0.03	0.29	80.20	
	Less than past year	11	**0.19**	0.08	0.30	78.1	
Alcohol use disorder	Race/ethnicity						0.33
	Black American	2	**0.23**	0.2	0.26	0	
	Latinx	2	**0.32**	0.15	0.48	95.20	
	Gender						0.45
	Male	2	**0.28**	0.04	0.51	98.50	
	Female	2	**0.18**	0.09	0.27	89.90	
Composite substance use	Study design						0.64
	Longitudinal	5	**0.15**	0.07	0.23	65	
	Cross‐sectional	14	**0.18**	0.1	0.26	89	
	Race/ethnicity						**0.003**
	Black American	7	**0.13**	0.09	0.18	62.80	
	Latinx	4	0.07	−0.17	0.31	91.30	
	Indigenous North American	3	**0.29**	0.23	0.35	0	
	Age group						**0.01**
	Young adult and adults	2	**0.25**	0.18	0.33	0	
	Youth and adolescents	10	**0.11**	0.03	0.19	83.90	
Smoking status	Study design						0.67
	Longitudinal	2	0.11	−0.04	0.27	70.70	
	Cross‐sectional	7	**0.16**	0.02	0.3	92.20	
	Race/ethnicity						0.5
	Black American	5	**0.2**	0.01	0.4	94.40	
	Latinx	2	0.1	−0.002	0.2	60.90	
	Asian American	2	**0.09**	0.03	0.14	0	
	Exposure timing						0.36
	Lifetime	3	0.27	−0.03	0.57	96.60	
	Past week	2	0.11	−0.04	0.27	70.70	
Presence–absence of alcohol use	Study design						0.55
	Longitudinal	3	**0.13**	0.04	0.22	81.10	
	Cross‐sectional	3	**0.17**	0.07	0.27	74.10	
Presence–absence of cannabis use	Study design						0.36
	Longitudinal	3	**0.09**	0.06	0.13	0	
	Cross‐sectional	4	**0.13**	0.05	0.21	74.20	
Presence–absence tobacco use	Study design						0.94
	Longitudinal	2	0.02	−0.14	0.55	98.30	
	Cross‐sectional	3	**0.22**	0.1	0.33	72.00	

*Note*: Bold values denote significance as per 95% CI's that do not include zero.

Abbreviations: CI LL, confidence interval lower limit; CI UL, confidence interval upper limit; k, number of effect sizes.

The results of the subgroup analyses can be found in Table 4. The majority of these analyses did not provide evidence for moderation effects. Yet across racial/ethnic groups, differences were found for tobacco use, with Black American and Latinx subgroups having comparable positive correlation coefficients, however, Indigenous North Americans had the strongest positive associations, and a negative association was observed for Asian Americans. Likewise, differences were also identified in the composite substance use outcome, with Indigenous North Americans again having the strongest associations, compared to Black Americans and Latinxs. Within the at‐risk/hazardous alcohol use domain, however, Black Americans had the strongest association, followed by Latinx and then Asian Americans.

Moderation by gender did not reveal any differences between male and female‐only samples across all AOD outcome domains.

In the alcohol use problems/consequences domain, adolescents and/or youths had stronger associations than young adults and/or adults. However, across the tobacco use, alcohol use, cannabis use, illicit substance use and binge drinking domains, there was a general trend of larger effect sizes in the young adult and/or adult subgroups.

Concerning moderation by study design, in the at‐risk/hazardous alcohol use and alcohol use problems/consequences domains, stronger associations were observed for cross‐sectional studies, compared to longitudinal studies.

Subgroup analysis by racial discrimination exposure timing demonstrated that, in the illicit substance use domain, lifetime exposure had a stronger association than past‐year exposure. Likewise, in the tobacco use domain, lifetime exposure also had the strongest positive association, followed by past year exposure and a negative association was observed for less than past year exposure.

## DISCUSSION

We used meta‐analytic methods to understand the association between racial and AOD outcomes. The findings suggest that there is considerable evidence to suggest that racial discrimination is a consistent correlate of distinct AOD outcomes in minoritised racial/ethnic groups, predominantly based in the United States. Across AOD outcomes, all the associations were in the small range [198], but varied in magnitude. This study also found preliminary evidence that race/ethnicity, age group, exposure timing and study design moderate these associations to some degree.

Given that most of the participants included within this review resided in the United States, it is likely that our findings mainly reflect the nature of racial discrimination and AOD in the United States context. Our findings, therefore, demonstrate that the United States’ history of racism continues to harm racially and ethnically minoritised groups residing in the country. However, considering all of the studies included in this review were conducted in post‐colonial and imperialist states/regions, the findings also point to enduring negative effects of these systems.

### Previous research

The findings of this study align with previous meta‐analyses, which report significant, positive associations between racial discrimination and AOD [35, 36, 199]. The magnitude of the associations between racial discrimination and alcohol use, binge drinking and alcohol use problems/consequences in the current study were similar to those reported by Desalu *et al*. [200], but the current study reports larger effect sizes for at‐risk/hazardous alcohol use and AUD. This may arise from the current study using the full Alcohol Use Disorder Identification Test (AUDIT) scale to measure at‐risk/hazardous alcohol use, whereas Desalu *et al*. [200] use the Alcohol Use Disorders Identification Test‐Consumption (AUDIT‐C). The full AUDIT captures health‐harming aspects of alcohol use, such as dependency and problems related to use, which have been previously demonstrated to have stronger associations with racial discrimination than use alone [95, 201, 202]. Moreover, despite both studies using the same operationalisation for AUD, differences in the racial discrimination‐AUD relationship across race/ethnicity might explain this discrepancy, as this study included a diverse minoritised racial/ethnic sample, while Desalu *et al*.'s [200] sample was Black American only.

Stronger associations between racial discrimination and at‐risk/hazardous alcohol use, alcohol problems and AUD compared to alcohol use have been reported in previous work [46, 93, 110, 202]. This suggests that more harmful alcohol outcomes are more closely related to racial discrimination, rather than just use. Substance use motives may explain this, as motives to avoid negative internal states are more related to maladaptive alcohol use, whereas social and enhancement motives are stronger predictors of use 32, 203, 204]. Unexpectedly, comparable associations between alcohol use and binge drinking were found, despite the unhealthy nature of binge drinking. Yet some evidence suggests that social motives are the strongest predictors of binge drinking [205, 206].

Our finding that racial discrimination had a weaker association with cannabis use, compared to other illicit substance use, is important. It demonstrates that incorporating cannabis into measures of illicit substance use in the context of racial discrimination is inappropriate and suggests that minoritised racial/ethnic groups exposed to racial discrimination may be at specific risk for illicit substance use. This finding corresponds to that reported by Garrett *et al*. [39] in their analysis of Cherokee Nation adolescents, which also observed that effect sizes for prescription and other illicit drug use were larger than for cannabis use. A recent analysis of diverse racial/ethnic groups (including White respondents) also reported larger effect sizes for the association between discrimination and illicit substances such as methamphetamines, than for cannabis, tobacco or alcohol, but smaller or comparable effect sizes for other illicit substances, including cocaine [207]. As the current study used a composite outcome for illicit substance use, the larger effect size may be driven by racial discrimination's stronger association with specific illicit substances. Yet it should be noted that, as the studies that contributed to both the illicit substance and cannabis use outcomes all used US‐based samples, these findings are likely only applicable to minoritised racial/ethnic groups based in the United States.

The finding that Indigenous North Americans appear to be at higher risk for tobacco use when exposed to racial discrimination compared to Black Americans, Latinxs and Asian Americans, may be because of the accessibility of tobacco, as Indigenous North Americans living on reservations report paying less for tobacco than Black Americans, Latinxs or Asian Americans [208] because of the exploitative marketing practices of tobacco companies [209, 210]. Therefore, the higher price point for tobacco products for other minoritised racial/ethnic groups may make the use of tobacco to cope with racial discrimination stress unviable. The similar finding observed for the composite substance use outcome suggests that Indigenous North Americans may be particularly vulnerable to the pernicious effects of racial discrimination. However, the current study is the first to document these findings, as studies that have assessed the moderating role of race/ethnicity in the racial discrimination‐AOD associations have consistently not included Indigenous North American participants.

The smaller effect sizes observed for Asian Americans, compared to Black Americans and Latinxs in the at‐risk/hazardous drinking domain, could be accounted for by the model minority myth (MMM). As there is some evidence to suggest that internalisation of the MMM impedes the ability to accurately perceive discrimination [211, 212], which may affect the degree to which discrimination is appraised as stressful. Accordingly, Asian Americans may be less likely to engage in maladaptive coping strategies, such as AOD, to cope with the stress of racial discrimination.

The larger effect sizes for young adults and/or adults across six of the AOD outcome domains, compared to adolescents and/or youths, may be accounted for by the simultaneous depletion of familial protective influences and increased exposure to racial discrimination, which have been documented to occur with aging into adulthood [213, 214, 215]. However, the opposite trend observed in the alcohol problems/consequences domain was unexpected. Notably, all the participants in the youth and/or adolescent subgroup for this outcome were Indigenous North American, who may be at particular risk for AOD when exposed to racial discrimination as discussed above. Therefore, this effect may be more driven by race/ethnicity than age.

The subgroup analyses identified that cross‐sectional studies produced larger effect sizes than longitudinal studies in the at‐risk/hazardous alcohol use and alcohol use problems domains. This finding aligns with Paradies *et al*. [216] meta‐analyses, where they noted that racism produced stronger effects on negative mental health in cross‐sectional studies, which has also been reported for minoritised ethnic groups living in the United Kingdom [217]. These findings suggest that the effects of racial discrimination may attenuate over time, potentially because of the progressive development of resiliency or the recruitment of protective resources, post‐exposure to racial discrimination. Alternatively, there is the possibility that the larger effect sizes observed for cross‐sectional studies reflect common‐method bias, which can inflate effect size estimates, as cross‐sectional studies are particularly vulnerable to this form of bias [218, 219, 220].

Our findings that lifetime exposure to racial discrimination had a stronger association with illicit substance and tobacco use, compared to recent exposure, are consistent with Carter *et al*.'s [36] results. Together, these findings support the weathering hypothesis [221], whereby as risk accumulates across the life course, so does the likelihood of negative health outcomes. Yet it is unclear why exposure timing is a significant moderator for some AOD outcomes and not others.

### Limitations

The present study should be interpreted in the context of some limitations. First, our subgroup analysis by race/ethnicity could only be conducted across broad racial categories. It was uncommon in the primary studies to capture data on distinct ethnic identities, and therefore, grouped participants were grouped primarily by the continent of heritage. This overlooks the substantial heterogeneity within these broad racial/ethnic categorisations. Therefore, future research should aim to collect detailed data on the ethnic identities of participants to facilitate more thorough investigations of how the racial discrimination‐AOD associations operate across distinct ethnic identities. Second, for some of the AOD outcomes, including at‐risk/hazardous cannabis use and cannabis use problems/consequences, only a small number of studies contributed to effect sizes and therefore should be interpreted with caution. Moreover, the low number of effect sizes available within subgroups, notably in the male and female‐only samples, likely limited our statistical power to detect moderation effects. Third, a small minority of studies included in this analysis were rated as good methodology quality, therefore, the inclusion of predominantly low to moderate‐quality studies can threaten the reliability and validity of these findings.

### Future research

Our findings have identified key areas for future research. First, the racial discrimination‐AOD relationships warrant further research in Indigenous North American populations, as this study provides preliminary evidence that they may be especially vulnerable to the impacts of racial discrimination. Second, as the vast majority of studies included in this review were based on US samples, research on this association in minoritised racial/ethnic groups outside of the United States is required. Third, this study suggests that future research should give considerably more attention to how the timing of racial discrimination impacts different AOD outcomes.

## CONCLUSIONS

In summary, the present study provides consistent evidence that racial discrimination is associated with AOD outcomes in minoritised racial/ethnic groups, yet this association varies to some degree across distinct AOD outcomes. We, therefore, provide evidence against the use of composite AOD measurements in this field. Moreover, the present study also suggests that these associations are somewhat modified by race/ethnicity, age, exposure timing and study design. The findings of this study could be informative for the development of prevention and intervention practices to mitigate the harmful effects of racism within minoritised racial/ethnic groups.

## AUTHOR CONTRIBUTIONS

**Evie Gates:** Conceptualization (equal); data curation (lead); formal analysis (lead); investigation (equal); methodology (equal); project administration (lead); validation (lead); writing—original draft (lead); writing—review and editing (lead). **Matthew Cant:** Data curation (supporting); investigation (supporting); methodology (supporting); project administration (supporting); validation (supporting); writing—review and editing (supporting). **Rebecca Elliott:** Conceptualization (equal); formal analysis (supporting); investigation (supporting); methodology (equal); supervision (lead); writing—original draft (supporting); writing—review and editing (supporting). **Patricia Irizar:** Data curation (supporting); investigation (supporting); methodology (supporting); project administration (supporting); supervision (supporting); writing—review and editing (supporting). **Christopher J. Armitage:** Conceptualization (equal); formal analysis (supporting); investigation (equal); methodology (equal); supervision (lead); writing—original draft (supporting); writing—review and editing (supporting).

## DECLARATION OF INTERESTS

None.

## CLINICAL TRIAL REGISTRATION

PROSPERO registration ID CRD42022381762 (https://www.crd.york.ac.uk/prospero/display_record.php?RecordID=381762)

## Supporting information


**Data S1.** Certainty asessment.

**Data S2.** Dependency criteria.

**Data S3.** Data.

**Data S4.** Forest plots.

**Data S5.** Sensitivity analysis.

## Data Availability

The data that support the findings of this study are available in supplementary materials 5.
