# Supplementary material for: A meta‐analytic review of the relationship between racial discrimination and alcohol and other drug use outcomes in minoritised racial/ethnic groups
Source: Addiction. 2025 Jul 16;120(12):2371–403. doi: 10.1111/add.70131 (PMC12586790; doi:10.1111/add.70131)
Supplement: Supplementary file 1 — Data S1. Certainty asessment. [file ADD-120-2371-s001.docx]

| **No of studies** | **Design** | **Risk of bias** | **Inconsistency** | **Indirectness** | **Imprecision** | **Publication bias** | **Certainty**  **(overall score)^^[[1]](#footnote-1)^^** |
| --- | --- | --- | --- | --- | --- | --- | --- |
| **Outcome:** Tobacco use | | | | | | | |
| 17 | Observational (Low ranking) | Not serious | Serious | Not serious | Serious | Not serious | 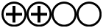 Low |
| **Outcome:** Alcohol use | | | | | | | |
| 41 | Observational (Low ranking) | Not serious | Serious | Not serious | Not serious | Not serious | 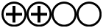 Low |
| **Outcome:** Cannabis use | | | | | | | |
| 20 | Observational (Low ranking) | Not serious | Serious | Not serious | Not serious | Not serious | 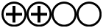 Low |
| **Outcome:** Illicit substance use | | | | | | | |
| 14 | Observational (Low ranking) | Not serious | Serious | Not serious | Not serious | Not serious | 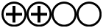 Low |
| **Outcome**: Binge drinking | | | | | | | |
| 15 | Observational (Low ranking) | Not serious | Not serious | Not serious | Not serious | Serious | 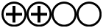 Low |
| **Outcome**: At-risk/hazardous alcohol use | | | | | | | |
| 29 | Observational (Low ranking) | Not serious | Serious | Not serious | Not serious | Not serious | 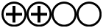 Low |
| **Outcome**: Alcohol use problems/consequences | | | | | | | |
| 23 | Observational (Low ranking) | Not serious | Not serious | Not serious | Not serious | Not serious | 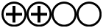 Low |
| **Outcome:** Substance use problems/consequences | | | | | | | |
| 5 | Observational (Low ranking) | Not serious | Serious | Not serious | Serious | Serious | Very low  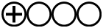 |
| **Outcome:** Alcohol use disorder | | | | | | | |
| 7 | Observational (Low ranking) | Not serious | Serious | Not serious | Not serious | Serious | 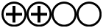 Low |
| **Outcome:** Substance use disorder | | | | | | | |
| 5 | Observational (Low ranking) | Not serious | Serious | Not serious | Serious | Serious | Very low 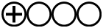 |
| **Outcome:** Composite substance use | | | | | | | |
| 19 | Observational (Low ranking) | Not serious | Serious | Not serious | Not serious | Not serious | 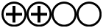 Low |
| **Outcome:** Smoking status | | | | | | | |
| 7 | Observational (Low ranking) | Serious | Serious | Not serious | Serious | Not serious | Very low 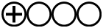 |
| **Outcome:** Presence – absence of alcohol use | | | | | | | |
| 6 | Observational (Low ranking) | Not serious | Serious | Not serious | Not serious | Serious | Very low 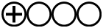 |
| **Outcome:** Presence – absence of tobacco use | | | | | | | |
| 5 | Observational (Low ranking) | Serious | Serious | Not serious | Serious | Serious | Very low 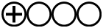 |
| **Outcome:** Presence – absence of cannabis use | | | | | | | |
| 7 | Observational (Low ranking) | Not serious | Not serious | Not serious | Not serious | Not serious | 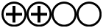 Low |
| **Outcome:** At-risk/hazardous cannabis use | | | | | | | |
| 4 | Observational (Low ranking) | Not serious | Not serious | Not serious | Serious | Serious | Very low 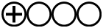 |
| **Outcome:** Cannabis use problems/consequences | | | | | | | |
| 2 | Observational (Low ranking) | Not serious | Not serious | Not serious | Serious | Serious | Very low 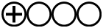 |

1. [↑](#footnote-ref-1)
