# Supplementary material for: A meta‐analytic review of the relationship between racial discrimination and alcohol and other drug use outcomes in minoritised racial/ethnic groups
Source: Addiction. 2025 Jul 16;120(12):2371–403. doi: 10.1111/add.70131 (PMC12586790; doi:10.1111/add.70131)
Supplement: Supplementary file 2 — Data S2. Dependency criteria. [file ADD-120-2371-s004.docx]

Supplementary materials 1 – Dependency management

Dependency across studies
Where multiple studies had utilised the same secondary data source, only one study was included in the meta-analyses to avoid violating assumptions of independence. The decision concerning which study was included was based on the following criteria: 1) studies with the largest sample size were given priority, 2) studies using longitudinal methods were prioritised over cross-sectional methods, 3) studies that perform subgroup/analysis stratified by ethnicity, age and gender were prioritised over studies which only conduct whole sample analyses. 4) Studies that were the most recently published/conducted were prioritised. 5) Studies that include discrete types/patterns of substance and/or alcohol use were prioritised over studies that analysed a composite substance use variable.

Dependency within studies
Samples/sub-samples were treated as the unit of analysis for the current study and treated as independent. Thus, instead of each study providing 1 effect size, each sample/sub-sample provides 1 effect size. I.e., if a study reports two individual effect sizes, one for African American respondents and one for Latinx respondents, these will be treated as independent. However, in cases where multiple effect sizes were reported for each sample/sub-sample per each substance and/or alcohol use outcome (i.e. two effect sizes are reported for the African American sample, one for the association between racial discrimination and alcohol consumption over the past 6 months, and one for the past 12 months) these will be dependent. As such, a prioritisation criterion was developed to identify which effect size per sample and per outcome should be included in the analysis: 1) In cases where both single-item and multiple-item racial discrimination and/or substance and alcohol use measures have been used, the multiple-item measure is prioritised. 2) In cases where both cross-sectional and longitudinal associations are reported, longitudinal ones were prioritised. 3) If multiple longitudinal effect sizes were reported, the one that covers the longest breadth of time was selected. 4) In cases where multiple effect sizes were reported for the same substance and/or alcohol use outcome or predictor but at different timeframes (i.e., past 6 months alcohol use, past 12 months alcohol use), the effect size that covers the longest time period was selected. 5). In cases where the racial discrimination predictor was converted to an ordinal scale (i.e., low, moderate and high) and analysed separately, the category that represents the highest ‘level’ of discrimination was prioritised. 6). In cases where the substance and/or alcohol use outcome was converted to an ordinal scale (i.e., used cocaine 1-10 times, 11-99 times, 100+ times), the category that represents the highest ‘level’ of use was selected. 7) In cases where a study captures direct and vicarious experiences of racial discrimination individually, the measure that captures direct experiences was prioritised. 8) In cases where a study captures multiple types of discrimination, i.e., from different sources. The measure that captures a broader range/general experiences of racial discrimination was prioritised. 9). In cases where the same AOD outcome is measured on a continuous scale and a nominal scale (i.e., frequency of alcohol use, any alcohol use), the effect size that corresponds with the continuous measurement of the outcome was prioritised. Except for diagnostic/clinical outcomes, where the binary outcome provides meaningful information. 10) If a study includes outcomes such as substance/alcohol abuse and dependence as well as substance use disorder, the use disorder outcome will be prioritised as it is in line with the most up-to-date diagnostic criteria. 11. In cases where multiple illicit drug use outcomes are modelled as separate outcomes, the illicit substance that is most frequently endorsed by the sample will be prioritised to be included in the illicit substance use outcome. 12. In cases where effect sizes are reported for the whole sample and relevant sub-samples, the effect sizes for sub-samples will be prioritised. In cases where a justification cannot be provided to prioritise one EF over another, the EF included in the analysis will be selected at random. The above criteria can be superseded if one of the dependent results has an available or convertible effect size.
