# Supplementary material for: A meta‐analytic review of the relationship between racial discrimination and alcohol and other drug use outcomes in minoritised racial/ethnic groups
Source: Addiction. 2025 Jul 16;120(12):2371–403. doi: 10.1111/add.70131 (PMC12586790; doi:10.1111/add.70131)
Supplement: Supplementary file 4 — Data S4. Forest plots. [file ADD-120-2371-s002.docx]

Supplementary materials 2 – Forest plots


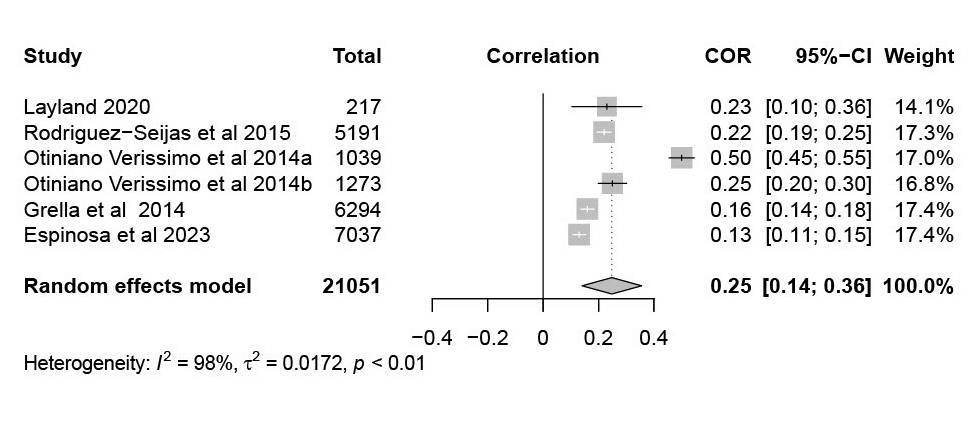
Substance use disorder forest plot
*Containing Pearson, Biserial and Tetrachoric correlations. I.e. outcome is measured continuously or is measured as a false dichotomy.

Substance use problems forest plot


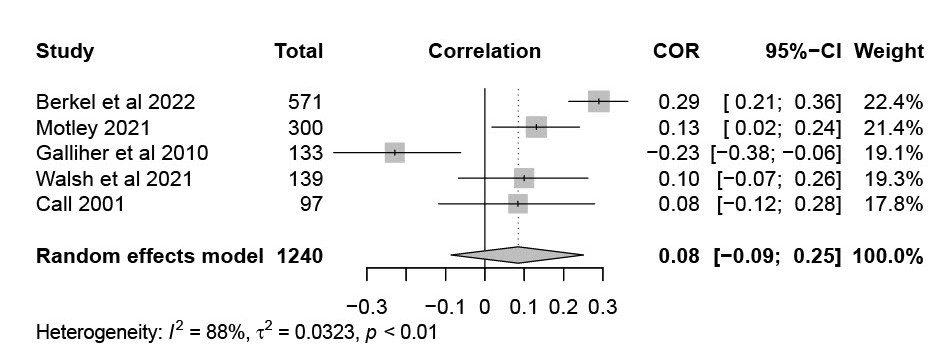


*Containing Pearson, Biserial and Tetrachoric correlations. I.e. outcome is measured continuously or is measured as a false dichotomy.


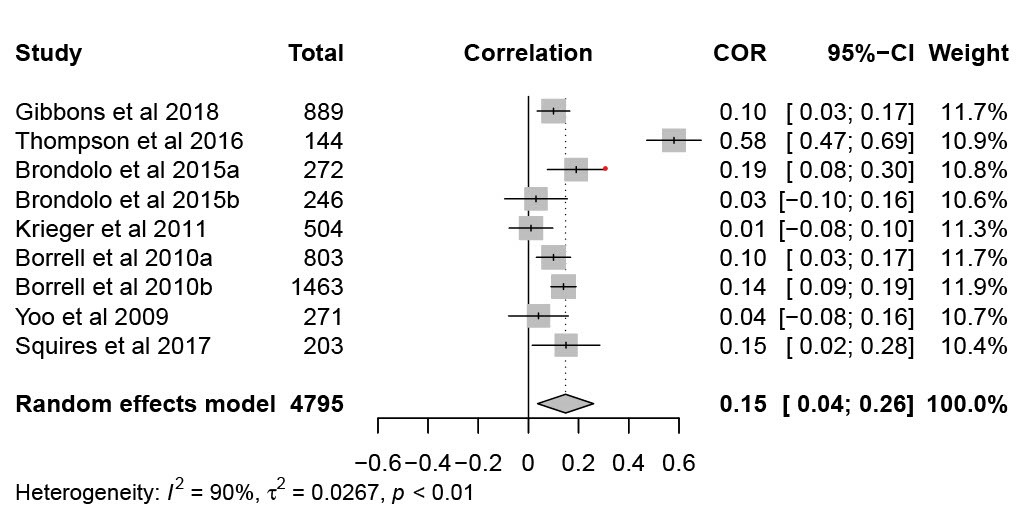
Smoking status forest plot

*Containing point-biserial correlations, i.e. outcomes represent true dichotomy


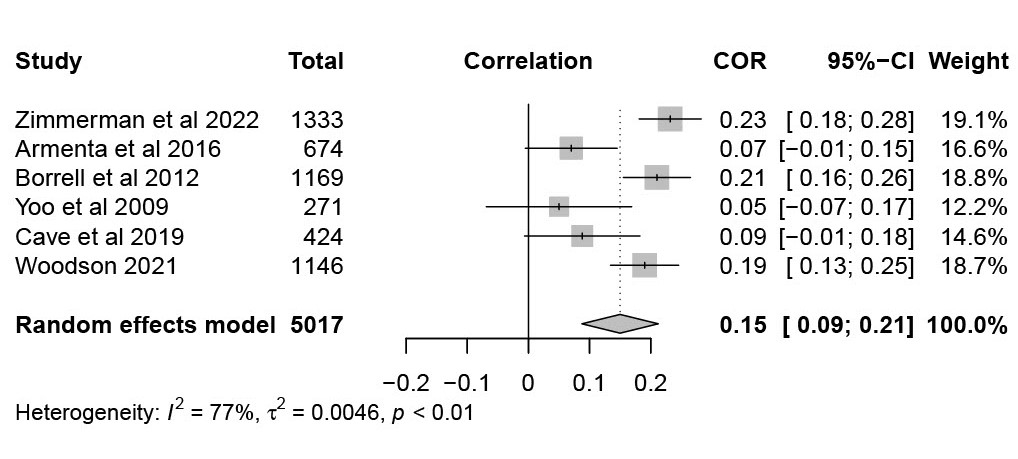
Presence – absence of alcohol use forest plot

*Containing point-biserial correlations, i.e. outcomes represent true dichotomy


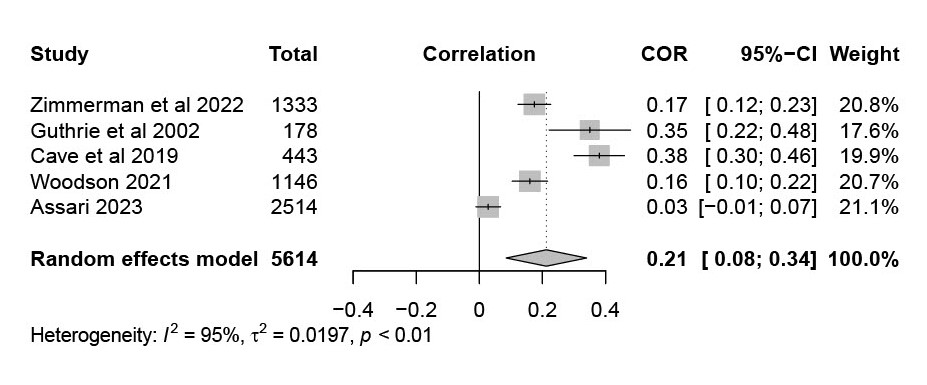
Presence – absence of tobacco use forest plot

*Containing point-biserial correlations, i.e. outcomes represent true dichotomy


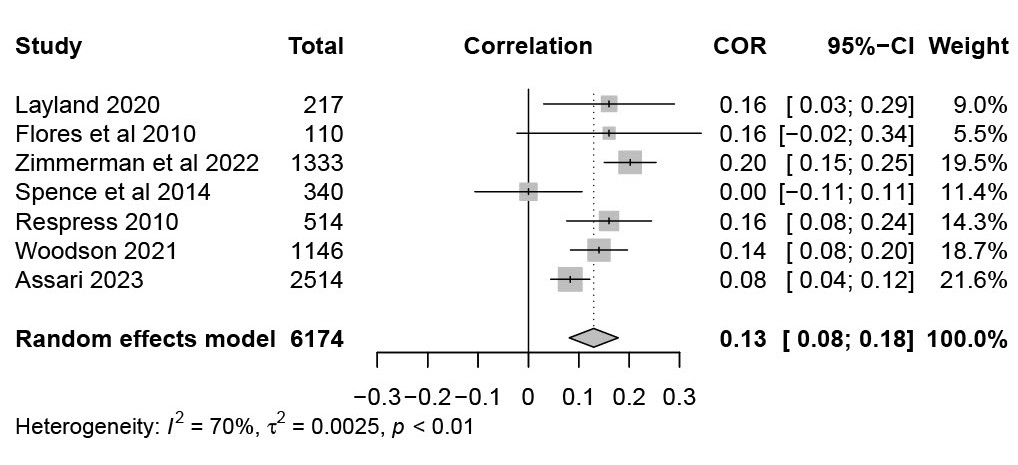
Presence – absence of cannabis use forest plot

*Containing point-biserial correlations, i.e. outcomes represent true dichotomy


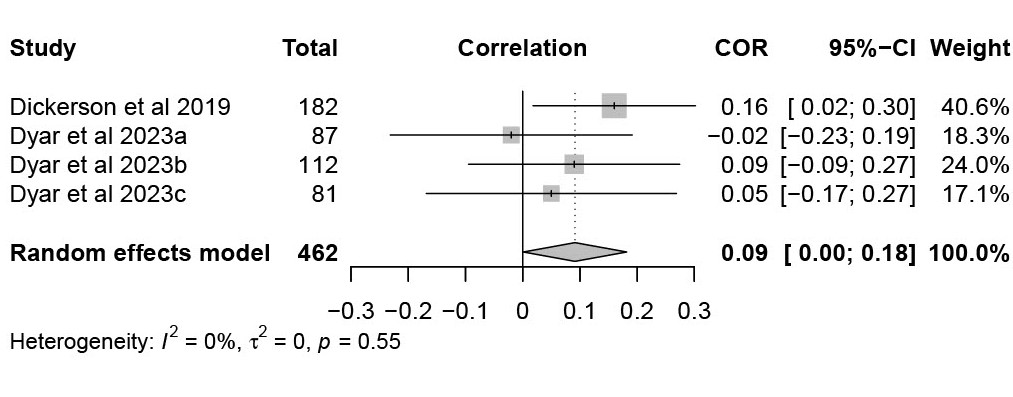
Cannabis use problems/consequences forest plot

*Containing Pearson, Biserial and Tetrachoric correlations. I.e. outcome is measured continuously or is measured as a false dichotomy.


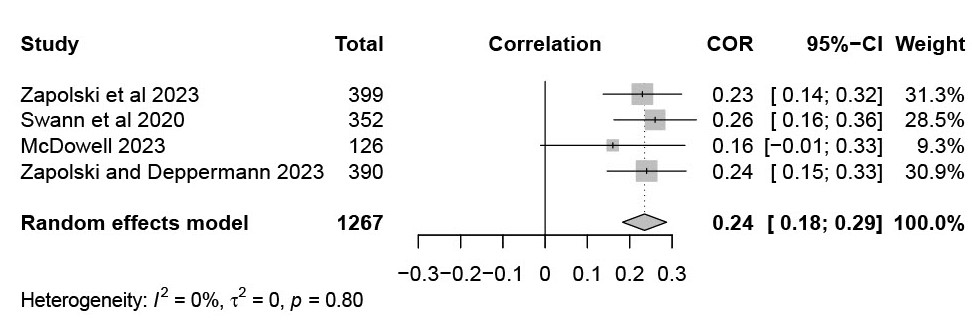
At-risk/hazardous cannabis use forest plot

*Containing Pearson, Biserial and Tetrachoric correlations. I.e. outcome is measured continuously or is measured as a false dichotomy.


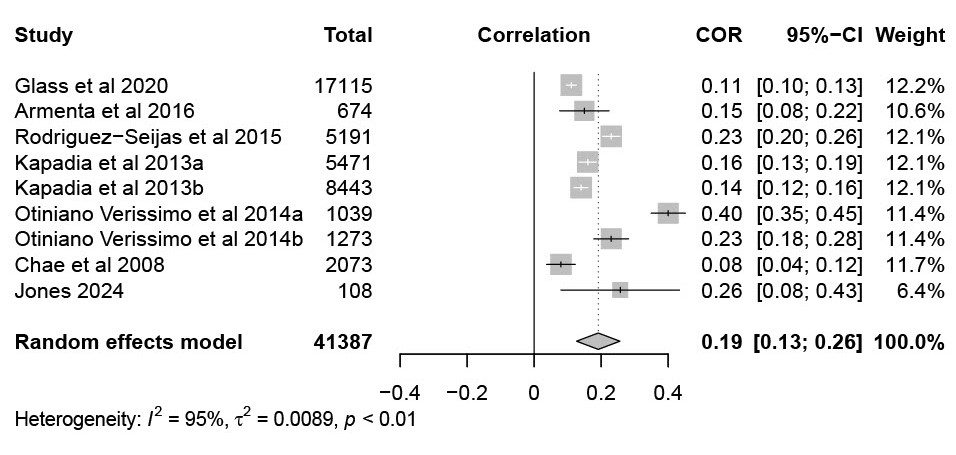
Alcohol use disorder forest plot

*Containing Pearson, Biserial and Tetrachoric correlations. I.e. outcome is measured continuously or is measured as a false dichotomy.


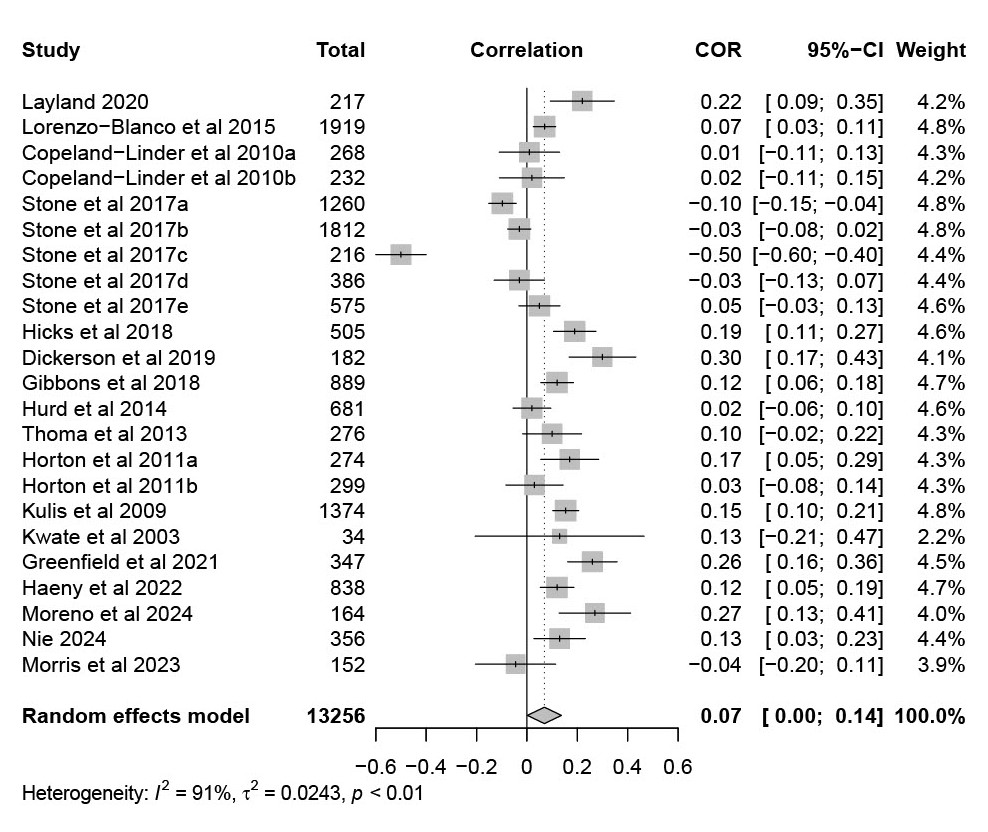
Tobacco use forest plot

*Containing Pearson, Biserial and Tetrachoric correlations. I.e. outcome is measured continuously or is measured as a false dichotomy.


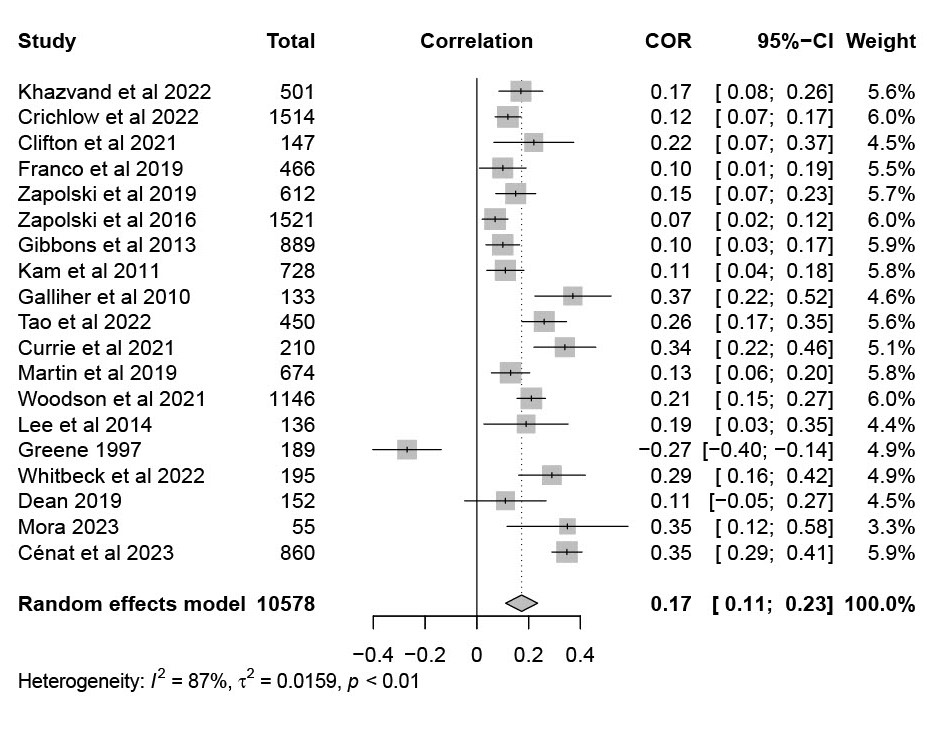
Composite substance use forest plot

*Containing Pearson, Biserial and Tetrachoric correlations. I.e. outcome is measured continuously or is measured as a false dichotomy.


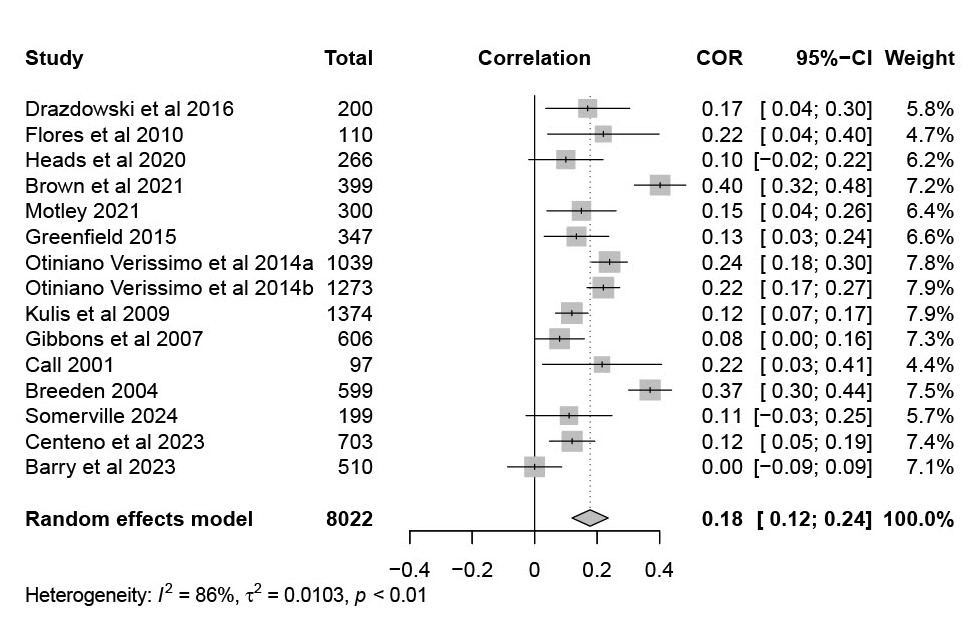
Illicit substance use forest plot

*Containing Pearson, Biserial and Tetrachoric correlations. I.e. outcome is measured continuously or is measured as a false dichotomy.


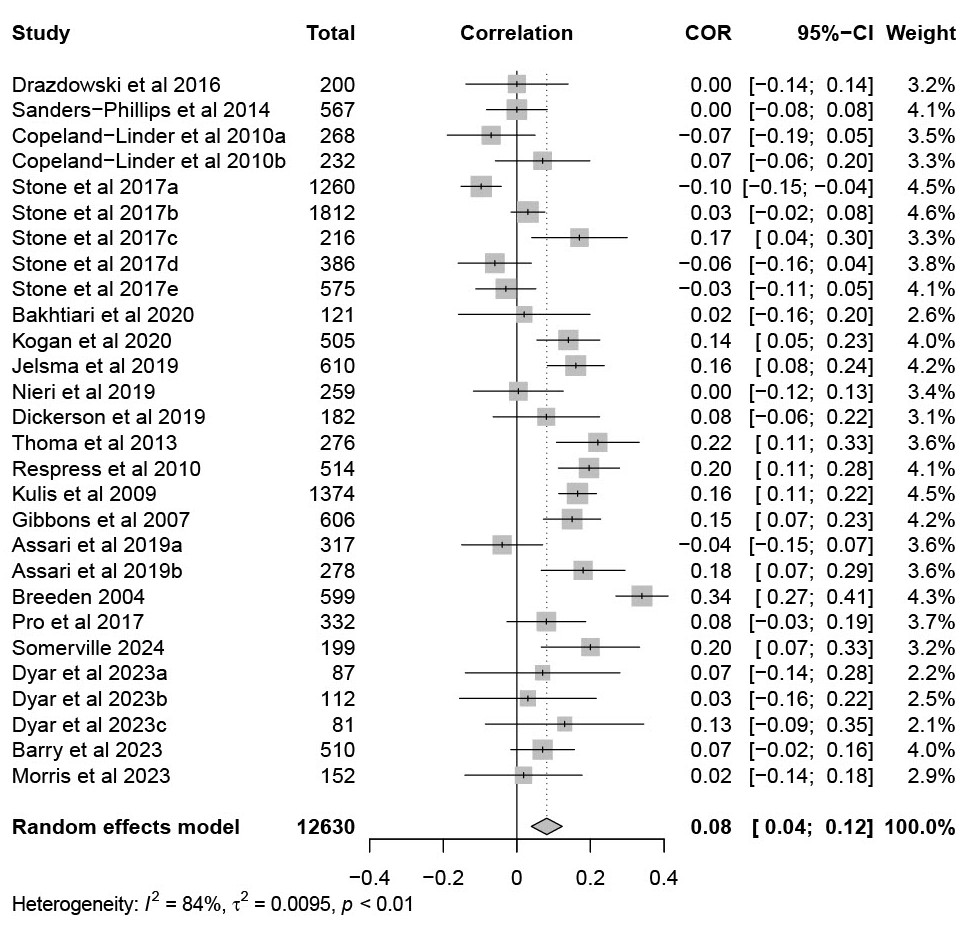
Cannabis use forest plot

*Containing Pearson, Biserial and Tetrachoric correlations. I.e. outcome is measured continuously or is measured as a false dichotomy.

Binge drinking forest plot


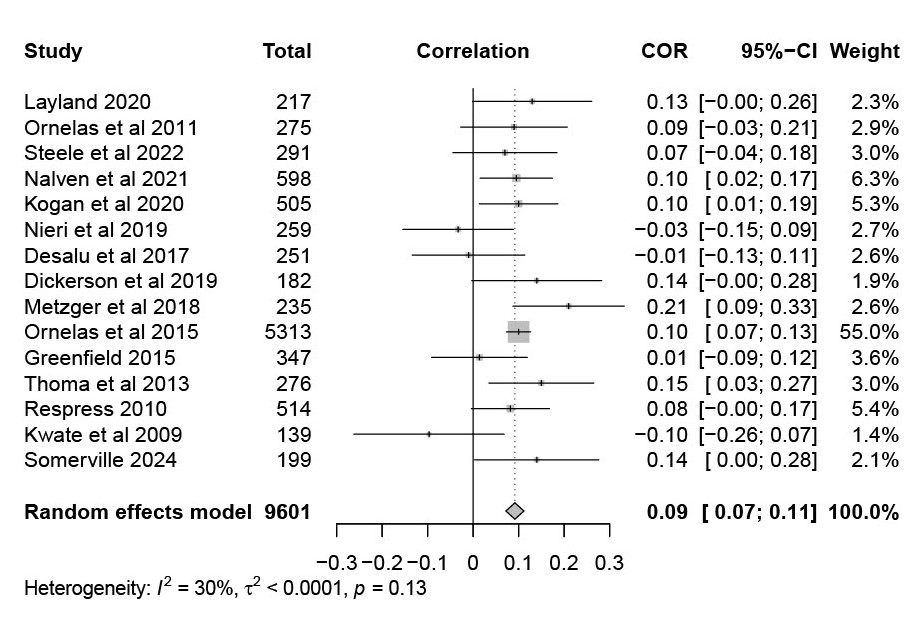


*Containing Pearson, Biserial and Tetrachoric correlations. I.e. outcome is measured continuously or is measured as a false dichotomy.


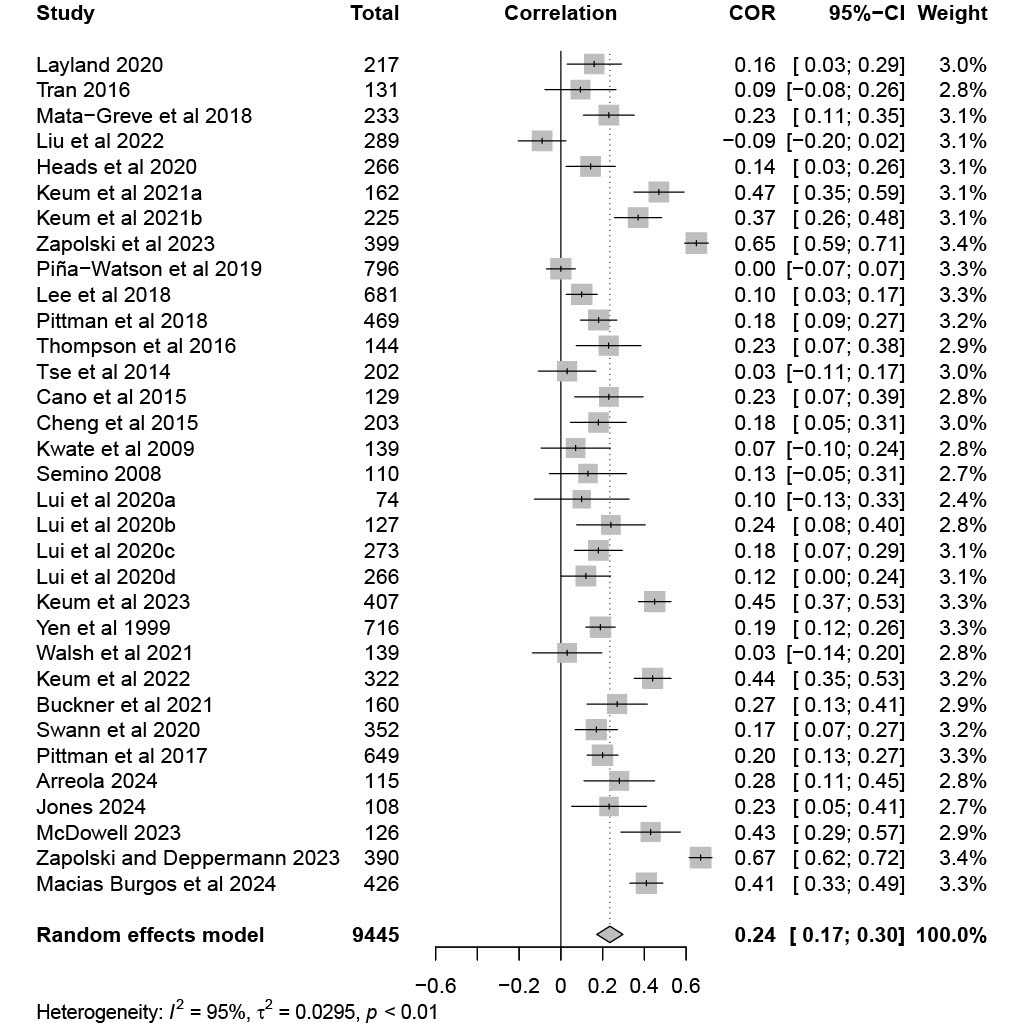
At-risk/hazardous alcohol use forest plot

*Containing Pearson, Biserial and Tetrachoric correlations. I.e. outcome is measured continuously or is measured as a false dichotomy.


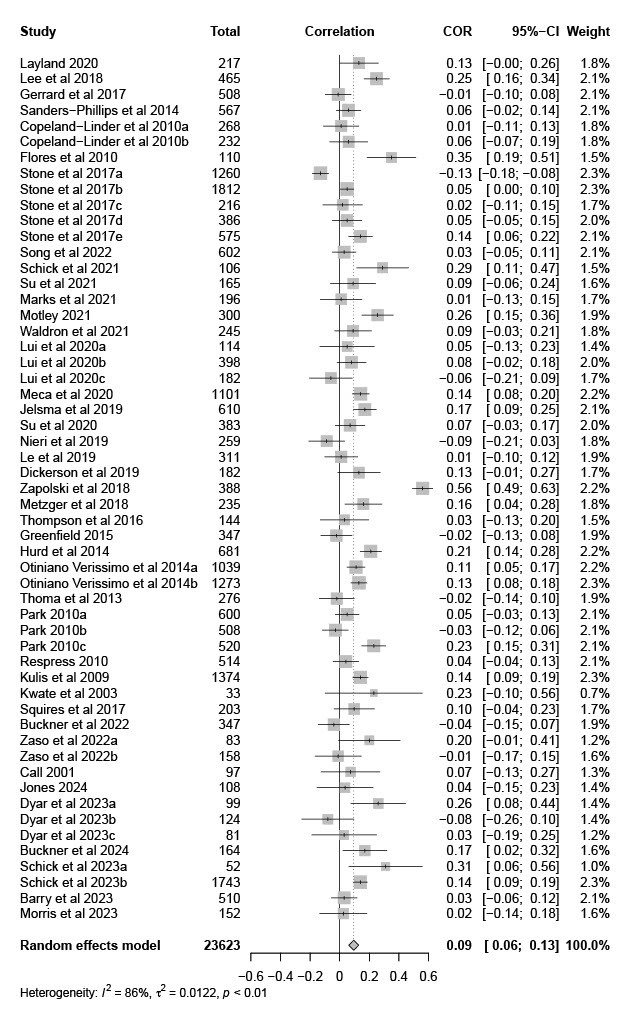
Alcohol use forest plot

*Containing Pearson, Biserial and Tetrachoric correlations. I.e. outcome is measured continuously or is measured as a false dichotomy.

Alcohol problems/consequences forest plot


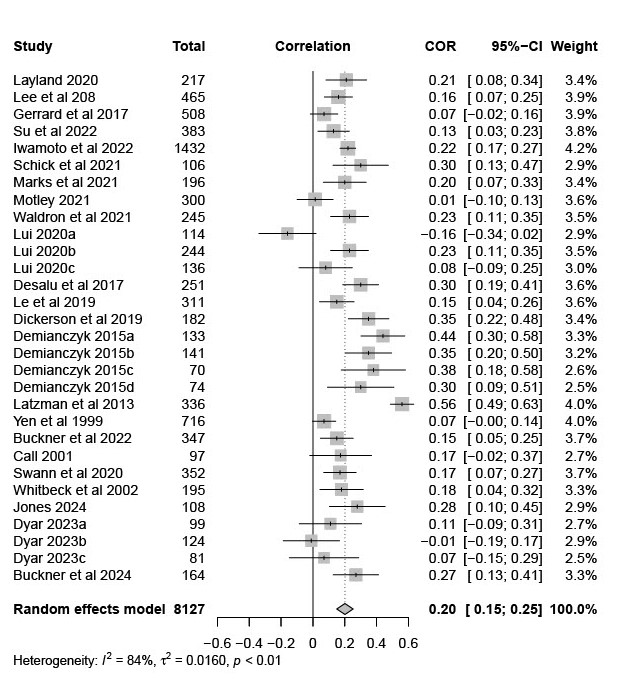


*Containing Pearson, Biserial and Tetrachoric correlations. I.e. outcome is measured continuously or is measured as a false dichotomy.
