# Supplementary material for: A meta‐analytic review of the relationship between racial discrimination and alcohol and other drug use outcomes in minoritised racial/ethnic groups
Source: Addiction. 2025 Jul 16;120(12):2371–403. doi: 10.1111/add.70131 (PMC12586790; doi:10.1111/add.70131)
Supplement: Supplementary file 5 — Data S5. Sensitivity analysis. [file ADD-120-2371-s005.docx]

*Sensitivity analysis*The main analysis was performed without the inclusion of studies rated as poor methodological quality to determine their impact on the pooled effect sizes. Significance was determined by confidence intervals that did not include zero.

Tobacco use
A significant positive correlation coefficient remained, which increased marginally. The degree of heterogeneity reduced (r = .11, 95% CI .06, .17, I² = 70.4%).

Alcohol use
A positive, slightly larger correlation coefficient was observed that remained statistically significant, and the degree of heterogeneity remained high (*r* = .13, *95% CI* = .08, .17, *I²* = 87.2%).

Cannabis use
A comparable positive correlation coefficient was observed which remained significant, however the degree of heterogeneity reduced (*r* = .09, *95% CI* = .04, .14, *I²* = 53.4%).

Illicit substance use
A significant, positive association remained but the coefficient reduced slightly, however heterogeneity reduced markedly (*r* = .17, *95% CI* = .12, .21, *I²* = 46%).

Binge drinking
A significant, positive association remained, and the correlation coefficient was unchanged, but heterogeneity decreased (r = .09, 95% CI = .06, .11, I² = 21.5%).

At-risk/hazardous alcohol use
A significant positive correlation remained, and the coefficient was largely unchanged. Likewise, heterogeneity was still considerable (r = .25, 95% CI = .15, .34, I² = 96.4%).

Alcohol use problems/consequences
A significant, positive correlation remained, with a slightly reduced coefficient. However, the degree of heterogeneity reduced substantially *(r* = .18, *95% CI* =.14, .22*, I²* = 54.1%).

Substance use problems/consequences
Sensitivity analysis was not possible for this outcome as all the included studies were rated as fair.

Alcohol use disorder
 A significant positive correlation remained, and the coefficient was unchanged. Likewise, heterogeneity remained high (*r* = .19, *95% CI* = .11, .26, *I²* = 94.9%).

Substance use disorder
A significant positive association remained, and the correlation coefficient was unchanged (*r* = .25, *95% CI* = .12, .39, *p* <.001). Moreover, the degree of heterogeneity also remained comparable (*Q* = 215.04, *p* <.001, *I²* = 98.1%).

Composite substance use
A significant positive association remained, and the correlation coefficient increased marginally, and heterogeneity remained high (*r* = .20, *95% CI* = .12, .28, *I²* = 86.3%).

Smoking status
A significant positive association remained; however, the coefficient had reduced. Likewise, the degree of heterogeneity dropped into the low to moderate range (r =.09, 95% CI = .03, .16, I² = 49.1%).

Presence – absence of alcohol use
A comparable, significant positive correlation remained. The degree of heterogeneity was largely unchanged (r = .17, 95% CI = .07, .27, I² = 74.1%).

Presence – absence of tobacco use
A comparable positive correlation coefficient was identified; however, the association was no longer significant. The degree of heterogeneity continued to be considerable (*r* = .20, *95% CI* = -.14, .55, *I²* = 98.3%).

Presence – absence of cannabis use
A significant, positive correlation remained but reduced in magnitude. The degree of heterogeneity also decreased (*r* = .09, *95% CI* = .02, .15, *I²* = 22.8%).

Cannabis use problems/consequences
Sensitivity analysis was not possible for this outcome as all the included studies were rated as fair.

At-risk/hazardous cannabis use
Sensitivity analysis was not possible for this outcome as all the included studies were rated as fair.

Publication bias
 In correspondence with the sensitivity analyses, a reassessment of publication bias on fair and good rated studies only was performed on outcomes where minor or major plot asymmetry was detected. In the binge drinking outcome, the Eggers test remained non-significant (p = 0.24), but the funnel plot became more asymmetric. Moreover, the doiplot and LFK index indicated major plot asymmetry (LFK = -2.33). For the composite substance use outcome, Eggers test on fair/good studies was not possible due to too few studies, and funnel plots remained largely unchanged, but doiplots and LFK index suggested major plot asymmetry (LFK = 2.67). In the at-risk/hazardous alcohol use outcome, the Eggers test remained significant (p = 0.01), funnel plots indicated minor asymmetry, and doiplots and LFK index suggested major plot asymmetry (LFK = -2.83). In the alcohol use disorder and substance use disorder outcomes, doiplots and LFK index continued to indicate major plot asymmetry (LFK = 3.13, 3.03, respectively). Re-analyses of publication bias was not possible for the cannabis problems/consequences, at-risk cannabis use, substance use problems presence – absence of alcohol use, and presence – absence of tobacco use outcomes, as these outcomes either contained studies which were all rated as fair or good, or when poor rated studies were removed, doiplots and the LFK index were under-powered to detect asymmetry.
